# Simple Estimators of the Mixing Proportion in a Semi-Parametric Mixture with Known Component

**DOI:** 10.1007/s13171-025-00421-w

**Published:** 2025-10-22

**Authors:** Fadoua Balabdaoui, Harald Besdziek

**Affiliations:** Seminar for Statistics, ETH Zürich, Zürich, Switzerland

**Keywords:** Asymptotic theory, Brownian process, Mixture distribution, Semi-parametric, Primary 62E20, Secondary 62F03, 62H30

## Abstract

In this paper, we consider the problem of estimating a mixture of a background and signal component. The background distribution is fully known, while the signal distribution needs to be estimated along with the mixing proportion. We treat the special case where the support of the signal distribution is strictly included in that of the background. We show how this assumption can be accounted for in the estimation procedure to obtain a parametric rate of convergence for estimating the mixing proportion. In the case where the signal distribution admits a monotone, a monotone and convex or a log-concave density with respect to Lebesgue measure, we construct estimators that are based on the well-known shape-constrained approaches adapted for each one of these cases. Simulations are presented to illustrate the obtained theoretical results. We also showcase our methodology using prostate cancer data.

**Supplementary Information:**

The online version contains supplementary material available at 10.1007/s13171-025-00421-w.

## Introduction

For a given (but unknown) probability $\alpha _0 \in (0,1)$, consider the two-component mixture model

1 $$\begin{aligned} F_0 = (1-\alpha _0) F_b + \alpha _0 F_s \end{aligned}$$

where $F_b$ and $F_s$ denote the background and signal distribution functions. The background distribution is completely known, while the mixing proportion as well as the signal distribution are to be estimated. This model has a long history and has been studied by several researchers; see Patra and Sen (2016) and the references therein. One important application of this model is multiple testing. In this problem, a given p-value can be either computed under the null hypothesis ($H_0$) or the alternative one ($H_1$). Thus, the resulting distribution is a mixture of a known distribution under $H_0$, e.g. the uniform distribution on [0, 1], and an unknown distribution under $H_1$, which needs to be estimated along with the mixing proportion. Other applications are related to contamination, which occurs for example in astronomy, where the main interest is to estimate the distribution of radial velocities of stars in some galaxy. For the dwarf galaxy Carina, the radial velocity measurements of its stars are contaminated with Milky Way stars in the field view. See (Patra and Sen, 2016 Section 9.2) for more details.

The main goal of this work is to suggest a simple estimator of the mixing proportion $\alpha _0$ that is fast to compute and also easy to use for inference purposes. Our main assumption is that the support of the signal distribution is *strictly* included in that of the background. More formally, let $\mu _b$ and $\mu _s$ be the Lebesgue-Stieltjes probability measures associated with the distribution functions $F_b$ and $F_s$ respectively. Let $\mathcal {S}_b$ and $\mathcal {S}_s$ denote the respective supports of $\mu _b$ and $\mu _s$, that is

$$\begin{aligned} \mathcal {S}_a = \overline{\{ B \in \mathcal {B}: \mu _a(B) > 0 \}}, \ \ \text {for}\ a \in \{b, s \}, \end{aligned}$$

where $\mathcal {B}$ denotes the Borelean $\sigma$-algebra on $\mathbb {R}$, and $\overline{C}$ the closure of some subset $C \subset \mathbb {R}$. In the whole paper, it will be assumed that

2 $$\begin{aligned} \mathcal {S}_s \subset \mathcal {S}_b \end{aligned}$$

where the inclusion is strict. For example, if $F_b = \Phi$ is the distribution of a standard Gaussian random variable, then the assumption in Eq. 2 is satisfied by any signal which has a $\text {Gamma}(\alpha , \beta )$ distribution for shape and rate $\alpha , \beta > 0$. Note that under this assumption, the mixture model in Eq. 1 is identifiable. To see this, note that we can find *x* such that $F_s(x) =0$ and $F_b(x) > 0$. This implies that $\alpha _0 = 1 - F_0(x)/F_b(x)$. In Patra and Sen (2016), the authors consider this setting as a corollary of their Lemma 4 under the additional assumption that $F_b$ and $F_s$ are absolutely continuous with densities $f_b$ and $f_s$ respectively. In fact, their Lemma 4 implies that if there exists no $c > 0$ such that $f_s(x) \ge c f_b(x)$ for almost every *x*, then identifiability holds. This condition is true under the assumption Eq. 2 because such an equality cannot hold for all *x* such that $f_s(x) =0$ and $f_b(x) > 0$.

It is worthwhile to note that the assumption Eq. 2 implies that it is impossible to have $\mathcal {S}_s = \mathbb {R}$. Thus, one can be in one of the following situations:

*1.*: There exists an unknown $a_0 \in \mathbb {R}$ such that $(-\infty , a_0) \subset \mathcal {S}^c_s$, and $(-\infty , a_0)$ is the largest set with this property,
*2.*: There exists an unknown $a_0 \in \mathbb {R}$ such that $(a_0, \infty ) \subset \mathcal {S}^c_s$, and $(a_0, \infty )$ is the largest set with this property,
*3.*: $\mathcal {S}_b = \mathbb {R}$ and there exist unknown $-\infty< a_1< a_2 < \infty$ such that $\mathcal {S}^c_s \subset (a_1, a_2)$ and $(a_1, a_2)$ is the largest interval with this property.
*4.*: $$\mathcal {S}_b = [b_1, b_2]$$, where $$- \infty \le b_1 < b_2 \le \infty $$ are known, and there exist unknown real numbers $$s_1 < s_2 $$ such that $$b_1 \le s_1 < s_2 \le b_2$$ and $$\mathcal {S}_s = [s_1, s_2]$$,

Some comments about these four settings are in order. Note that in Problem #1 (resp. #2), we only know that the signal distribution is not supported on $(-\infty , a_0)$ (resp. $(a_0, \infty )$) and that this interval is the largest with such a property. Hence, this does not exclude for instance the possibility that the signal distribution is not supported on some interval [*b*, *c*] for $a_0< b < c \le \infty$ (resp. $- \infty \le b< c < a_0$ ). For example, consider the case where the background distribution is Gaussian and the signal distribution is Uniform on [0, 1]. Suppose also that all we know is that the signal distribution is not supported on $(-\infty , a_0)$. Then, in this case illustrating Problem #1, we have that the true $a_0 =0$ (to be estimated) and $(-\infty , 0)$ is the largest interval on which the signal distribution is not supported. However, this distribution is not supported on $(1, \infty )$ either. As this information is not available, it is not relevant here as it cannot be used in the estimation procedure. Let us consider another example where the background distribution is now Uniform on [0, 1], the signal distribution is Uniform on [0, 0.6] and we only know that it is not supported on $(a_0, \infty )$ (here the true $a_0 = 0.6$). Although the signal distribution is not supported on $(-\infty , 0)$, this information is not revealed and hence does not play any role in estimating $\alpha _0$. The settings of Problem #3 and #4 give more information about the nature of the support of the signal, although Problem #3 presents some serious challenges; see Section 5. Note that the second example provided above fits well in Problem #4 where, with $b_1 = 0, b_2 = 1$, the information that the signal distribution is not supported on $(-\infty , 0)$ is now available and can be used in the estimation of $\alpha _0$.

In this work, we are going to focus on Problem #1 and Problem #2 where less information is available. Although estimation in Problem #3 and #4 is also very worth investigating, we cannot do this here in full length and provide instead some insights in Section 5.

In the sequel, it will be assumed that $F_s$ is continuous on $\mathbb {R}$. Under either setting of Problem #1 or #2, this continuity implies that $F_s(a_0) =0$. Note that going from Problem #1 to #2 can be done for example by resorting to mirroring the data, for example through multiplication by $-1$. For this reason, we will mainly develop the methodology for Problem #1.

The main goal of this paper is to consider new estimators of the unknown mixing proportion $\alpha _0$ under assumption Eq. 2 which are fast to compute even for very large sample sizes and which also enable meaningful inference such as building confidence intervals for $\alpha _0$ with the right asymptotic level. Our estimators turn out to converge to the truth at the parametric rate $1/\sqrt{n}$; i.e., the random estimation error decays like $1/\sqrt{n}$, with *n* the number of observations from the mixed distribution $F_0$. In comparison, Patra and Sen (2016) introduced estimators for $\alpha _0$ whose rate of convergence is $c_n/\sqrt{n}$, with $c_n = o(\sqrt{n})$ and $\lim _{n \rightarrow \infty } c_n = \infty$. The setting considered in Patra and Sen (2016) is more general, and hence it is expected that the convergence rate can be improved upon when additional information is used in the estimation procedure. This is the case in this work where the support of the signal distribution is known to be strictly included in that of the background. For the theoretical derivations, we shall focus on Problem #1, but we will consider Problem #2 as well in the simulations section. As the main obstacle is lack of knowledge of $a_0$, we will need to account for this missing information while constructing our estimators. Once $\alpha _0$ is estimated, we will show how we can consistently estimate $a_0$. Additionally, we will treat the problem of estimating the signal distribution under different shape hypotheses on its density (assumed to exist).

The manuscript will be organized as follows: In Section 2 we present the estimation method and give a weak convergence theorem for the constructed estimator. In particular, it will be shown that the convergence rate is parametric. In Section 3 we treat the problem of estimating $a_0$ and the signal distribution. Assuming that the latter is absolutely continuous, we consider estimation of the density of the signal under the following shape constraints: (i) decreasing monotonicity, (ii) decreasing monotonicity and convexity and (iii) log-concavity. Results of several simulations are presented in Section 4. We finish the paper with Section 5 where we gather some conclusions and discuss future directions for research in this important area of Statistics.

## Estimation of the Mixing Proportion

Consider the mixture distribution $F_0$ as in Eq. 1 and $X_1, \ldots , X_n$ i.i.d. random variables distributed according to $F_0$. In the following we make the assumptions

- **Assumption A1:** $F_b$ and $F_s$ are continuous
- **Assumption A2:** We are in the situation of Problem #1; i.e., $(-\infty , a_0) \subset \mathcal {S}^c_s$ for some $a_0$ and $(-\infty , a_0)$ is the largest set with this property.

Then, for all *x* such that $F_b(x) > 0$ it holds that

$$\begin{aligned} 1- \frac{F_0(x)}{F_b(x)} = \alpha _0 - \alpha _0 \frac{F_s(x)}{F_b(x)}, \end{aligned}$$

which in turn implies that

3 $$\begin{aligned} \sup _{ F_b(x)> 0} \left( 1- \frac{F_0(x)}{F_b(x)}\right) = \alpha _0 - \alpha _0 \inf _{F_b(x) > 0} \frac{F_s(x)}{F_b(x)} = \alpha _0. \end{aligned}$$

The identity above is an immediate consequence of the fact that the ratio $F_s/F_b$ is always non-negative and takes value exactly 0 when $x \notin \mathcal {S}_s$. Note that Assumption A2 implies that the support of the signal distribution is included in $[a_0, \infty )$. The identity in Eq. 3 is the starting point for constructing a new estimator. In fact, $F_b$ is known and $F_0$ can be estimated using the empirical cdf, $\mathbb {F}_n$, based on the observed sample $(X_1, \ldots , X_n$). Thus, it seems natural to consider the estimator

$$\begin{aligned} 1 - \inf _{F_b(x) > 0} \frac{\mathbb {F}_n(x)}{F_b(x)}. \end{aligned}$$

However, this estimator is not a good one. Indeed, it is always equal to 1 since the second term is 0 for values *x* that are strictly smaller than $X_{(1)} = \min _{1 \le i \le n} X_i$. Actually, even if we restrict the domain over which we take the infimum to $[X_{(1)}, \infty )$, the resulting estimator will still fail to be consistent. To see this, assume without loss of generality that $\alpha _0 \le 1/2$. Then, with probability tending to 1 (actually equal to $1-(1- F_0(a_0))^n)$, at least one observation will occur to the left of $a_0$. This means with probability tending to 1, we have that $F_0(X_{(1)})= (1-\alpha _0) F_b(X_{(1)})$. Then,

$$\begin{aligned} 1-\frac{\mathbb {F}_n(X_{(1)})}{F_b(X_{(1)})} = 1- \frac{1-\alpha _0}{n F_0(X_{(1)})}. \end{aligned}$$

Using the fact that

$$\begin{aligned} 1-\frac{\mathbb {F}_n(X_{(1)})}{F_b(X_{(1)})} \le 1- \inf _{ x \ge X_{(1)}} \frac{\mathbb {F}_n(x)}{F_b(x)} \end{aligned}$$

it follows that

$$\begin{aligned} P\left( 1 - \inf _{x \ge X_{(1)}} \frac{\mathbb {F}_n(x)}{F_b(x)}> \frac{3 \alpha _0}{2} \right) \ge P\left( 1- \frac{1-\alpha _0}{n F_0(X_{(1)})} > \frac{3 \alpha _0}{2} \right) . \end{aligned}$$

Using the well-known result that $F_0(X_{(1)}) \sim \text {Beta}(1, n)$, we can write

$$\begin{aligned} P\left( 1- \frac{1-\alpha _0}{n F_0(X_{(1)})}> \frac{3 \alpha _0}{2} \right)= &  P\left( F_0(X_{(1)})> \frac{1-\alpha _0}{n(1-3\alpha _0/2)} \right) \\= &  \left( 1 - \frac{1-\alpha _0}{n(1-3\alpha _0/2)} \right) ^n \\\rightarrow &  \exp \left( -\frac{1-\alpha _0}{1-3\alpha _0/2} \right) > 0 \end{aligned}$$

as $n \rightarrow \infty$, and hence

$$\begin{aligned} \liminf _{n \rightarrow \infty } P\left( 1 - \inf _{x \ge X_{(1)}} \frac{\mathbb {F}_n(x)}{F_b(x)}> \frac{3 \alpha _0}{2} \right) > 0. \end{aligned}$$

Recall that in constructing a new estimator, we use our knowledge that the support of the signal distribution is included in that of the background distribution and that the support of the signal starts to the right of some unknown $a_0$. Now, suppose that we know that $a_0 > \kappa _0$ for some fixed $\kappa _0$ such that $F_b(\kappa _0) > 0$.

Consider now the estimator

4 $$\begin{aligned} \hat{\alpha }_n = 1 - \inf _{x \ge \kappa _0} \frac{\mathbb {F}_n(x)}{F_b(x)}. \end{aligned}$$

In the following, we give our first main theorem.

### Theorem 2.1

Let $\mathbb {B}$ denote a standard Brownian bridge. Then, with $\hat{\alpha }_n$ and $\kappa _0$ defined as above, we have that

$$\begin{aligned} \sqrt{n} (\hat{\alpha }_n - \alpha _0) \rightarrow _d -\inf _{x \in [\kappa _0, a_0]} \frac{\mathbb {B} \circ F_0(x)}{F_b(x)}. \end{aligned}$$

A proof can be found in the supplementary material.

## Appropriateness of a Given $\kappa _0$ and Estimation of $a_0$

We start this section with estimation of $a_0$. This means that we will leave the question about deciding whether a certain $\kappa _0$ is appropriate to the next one.

### Estimation of $a_0$

In the sequel, we keep the same scheme as above. This means that we take for granted that Assumptions A1 and A2 hold. Just by definition, we have that

$$\begin{aligned} a_0= &  \sup \{x \ge \kappa _0: F_0(x) - (1-\alpha _0) F_b(x) = 0 \} \\= &  \sup \{x \ge \kappa _0: F_0(x) - (1-\alpha _0) F_b(x) \le 0 \}. \end{aligned}$$

#### Remark 1

Before we introduce our estimator for $a_0$, let us first discuss some properties of the signal cdf $F_s$. By definition of $a_0$ and Assumption A1 (continuity of $F_s$), we know that there exists a (possibly very small) $\eta > 0$ such that $F_s$ is strictly increasing on the interval $[a_0, a_0 + \eta ]$. On this interval, it is a homeomorphism, i.e., it has a continuous inverse $F_s^{-1}$. In addition, $F_s$ is always differentiable on the interval $(-\infty ,a_0)$ just because it is zero on the whole interval (in fact, it is even smooth there). Hence, the left difference quotient of $F_s$ at the point $a_0$ always exists and is equal to zero. So if the right difference quotient of $F_s$ at $a_0$ exists and has value zero, too, then we know that $F_s$ is differentiable at $a_0$.

Keeping these properties of $F_s$ in mind, we are now ready to introduce our estimator $\widehat{a}_n$ for $a_0$ which turns out to have really nice properties.

#### Theorem 3.1

Let $(b_n)_{n \ge 1}$ be a non-negative sequence such that

$$\begin{aligned} \lim _{n \rightarrow \infty } b_n = \infty , \ \ \ \text {and} \ \ \ b_n = o(\sqrt{n}). \end{aligned}$$

Consider the estimator

5 $$\begin{aligned} \widehat{a}_n = \sup \left\{ x \ge \kappa _0: \mathbb {F}_n(x) - (1-\widehat{\alpha }_n) F_b(x) \le \widehat{\alpha }_n \frac{b_n}{\sqrt{n}}\right\} . \end{aligned}$$

Let $\eta > 0$ be the same as in the remark above, i.e., $F_s$ is strictly increasing on the interval $[a_0, a_0 + \eta ]$.

Then, as $n \nearrow \infty$, we obtain the following:

**I.** Assume that $F_s$ is differentiable on the whole interval $(a_0,a_0 + \eta )$. Suppose that the right difference quotient of $F_s$ at the point $a_0$, denoted by $f_s(a_0)$, exists and that it is non-zero. Then, we have the convergence in probability

$$\begin{aligned} \frac{\sqrt{n}}{b_n} \Big (\widehat{a}_n - a_0\Big ) \overset{\mathbb {P}}{\rightarrow } \frac{1}{f_s(a_0)}. \end{aligned}$$

**II.** Suppose that $F_s$ is $k-1$ times continuously differentiable on the interval $[a_0,a_0 + \eta )$, for some integer $k \ge 2$ and with all $k-1$ derivatives being equal to zero. Denote the *j*-th derivative of $F_s$ by $F_s^{(j)},1 \le j \le k-1$. Assume that $F_s^{(k-1)}$ is continuously differentiable on the interval $(a_0,a_0 + \eta )$. Further suppose that the right difference quotient of $F_s^{(k-1)}$ at $a_0$, denoted by $F_s^{(k)}(a_0)_+$, exists and that it is non-zero. Then, we have the convergence in probability

$$\begin{aligned} \Big (\frac{\sqrt{n}}{b_n}\Big )^{1/k} \Big (\widehat{a}_n - a_0\Big ) \overset{\mathbb {P}}{\rightarrow } \Bigg (\frac{k!}{F_s^{(k)}(a_0)_+}\Bigg )^{1/k}. \end{aligned}$$

**III.** Assume that $F_s$ is differentiable on the whole interval $(a_0,a_0 + \eta )$ but that the right difference quotient of $F_s$ at $a_0$ is infinite. Then, we have the convergence in probability

$$\begin{aligned} \frac{\sqrt{n}}{b_n} \Big (\widehat{a}_n - a_0\Big ) \overset{\mathbb {P}}{\rightarrow } 0. \end{aligned}$$

A proof of the theorem above can be found in the supplementary material. In the first case, our estimator $\widehat{a}_n$ has a convergence rate of $\sqrt{n}/b_n$, which can be made arbitrarily close to $\sqrt{n}$ by choosing the sequence $(b_n)_{n \ge 1}$ appropriately. In the second case, we get a slower rate of convergence which depends on the integer *k*. In the third case, the rate is even faster than in the first case. Of course, our estimator $\widehat{a}_n$ is in all three cases consistent.

### Testing Whether a Given $\kappa _0$ is Appropriate

One of the main questions to be asked is how to decide whether a given $\kappa _0$ satisfies $\kappa _0 \le a_0$. The problem can be put in a formal hypothesis testing scheme. Consider the testing problem

$$\begin{aligned} H_0: \kappa _0 \le a_0 \ \ \ \text {versus} \ \ \ H_1: \kappa _0 > a_0. \end{aligned}$$

Under $H_0$, we have $F_s(x)=0$ for all $x \le \kappa _0$ which is also equivalent to writing that

$$\begin{aligned} \frac{F_0(x)}{F_b(x)} = 1-\alpha _0, \ \ \text {for all}\ x \le \kappa _0. \end{aligned}$$

Then, for all $x \le \kappa _0$

$$\begin{aligned} \frac{\mathbb {F}_n(x)}{F_b(x)}= &  \frac{\mathbb {F}_n(x) - F_0(x)}{F_b(x)} + 1-\alpha _0 \\= &  \frac{\mathbb {F}_n(x) - F_0(x)}{F_b(x)} + \frac{F_0(\kappa _0)}{F_b(\kappa _0)} \\= &  \frac{\mathbb {F}_n(x) - F_0(x)}{F_b(x)} - \frac{\mathbb {F}_n(\kappa _0) - F_0(\kappa _0)}{F_b(\kappa _0)} + \frac{\mathbb {F}_n(\kappa _0)}{F_b(\kappa _0)} \end{aligned}$$

which yields

6 $$\begin{aligned} \frac{\mathbb {F}_n(x)}{F_b(x)} - \frac{\mathbb {F}_n(\kappa _0)}{F_b(\kappa _0)} = \frac{\mathbb {F}_n(x) - F_0(x)}{F_b(x)} - \frac{\mathbb {F}_n(\kappa _0) - F_0(\kappa _0)}{F_b(\kappa _0)} \end{aligned}$$

for all $x \le \kappa _0$, and in particular for $x \in [\iota _0, \kappa _0]$ for a given $\iota _0 < \kappa _0$ such that $F_b(\iota _0) > 0$. Using standard arguments, it follows from Eq. 6 that the process

$$ \left\{ \frac{\mathbb {F}_n(x)}{F_b(x)} - \frac{\mathbb {F}_n(\kappa _0)}{F_b(\kappa _0)}, \ x \in [\iota _0, \kappa _0] \right\} $$

admits a weak limit after rescaling:

7 $$\begin{aligned} \left\{ \sqrt{n} \left( \frac{\mathbb {F}_n(x)}{F_b(x)} - \frac{\mathbb {F}_n(\kappa _0)}{F_b(\kappa _0)} \right) , \ x \in [\iota _0, \kappa _0] \right\} \nonumber \\ \Rightarrow \left\{ \frac{\mathbb {B} \circ F_0(x)}{F_b(x)} - \frac{\mathbb {B} \circ F_0(\kappa _0)}{F_b(\kappa _0)}, \ x \in [\iota _0, \kappa _0] \right\} . \end{aligned}$$

Different statistics can be based on the previous weak convergence to test $H_0$ versus $H_1$. Consider

$$\begin{aligned} T_n(\iota _0, \kappa _0):= \sup _{x \in [\iota _0, \kappa _0]} \sqrt{n} \left| \frac{\mathbb {F}_n(x)}{F_b(x)} - \frac{\mathbb {F}_n(\kappa _0)}{F_b(\kappa _0)} \right| . \end{aligned}$$

The convergence in Eq. 7 and the continuous mapping theorem imply that

$$\begin{aligned} T_n(\iota _0, \kappa _0) \rightarrow _d \sup _{x \in [\iota _0, \kappa _0]} \left| \frac{\mathbb {B} \circ F_0(x)}{F_b(x)} - \frac{\mathbb {B} \circ F_0(\kappa _0)}{F_b(\kappa _0)} \right| =: \mathbb {T}_\infty (\iota _0, \kappa _0). \end{aligned}$$

Obtaining a very good approximation of the quantiles of $\mathbb {T}_\infty (\iota _0, \kappa _0)$ can be done by approximating the distribution of the Brownian Bridge $\mathbb {B} \circ F_0$. One can appeal to the well-known Haar construction (see Pyke, 1983). Alternatively, we use the fact that $\{\mathbb {B}(t), t \in [0,1] \}$ is the weak limit as $N \nearrow \infty$ of $\{\widetilde{\mathbb {B}}_N(t):= \sqrt{N} \big (\mathbb {G}_N(t) - t\big ), t \in [0,1] \}$, where $\mathbb {G}_N = N^{-1} \sum _{j=1}^N \mathbb {I}_{\{ U_j \le t \}}$ with $U_1, \ldots , U_N$ i.i.d. $\sim \mathcal {U}[0,1]$, and that the empirical distribution $\mathbb {F}_n$ is a good surrogate for $F_0$. Then, taking $N = n$, one can compute the empirical upper quantiles of

8 $$\begin{aligned} \mathcal {T}_n(\iota _0, \kappa _0):= \sup _{x \in [\iota _0, \kappa _0]} \left| \frac{\widetilde{\mathbb {B}}_n \circ \mathbb {F}_n(x)}{F_b(x)} - \frac{\widetilde{\mathbb {B}}_n \circ \mathbb {F}_n(\kappa _0)}{F_b(\kappa _0)} \right| \end{aligned}$$

by generating *M* independent processes $\widetilde{\mathbb {B}}_n$. For a small $\beta \in (0,1)$, let $\tilde{q}_{1-\beta } = \tilde{q}_{1-\beta }(\iota _0,\kappa _0)$ denote the empirical quantile of order $1-\beta$. The null hypothesis $H_0: \kappa _0 \le a_0$ is accepted at the level $\beta$ if $T_n(\iota _0, \kappa _0) \le \tilde{q}_{1-\beta }$ (note that $\tilde{q}_{1-\beta }$ depends on *n* and *M*).

### From Problem #1 to #2

As mentioned in the introduction, going from Problem #1 to Problem #2 can be done through multiplying the data with $-1$. Under the Assumption A1, the distribution function of $- X$ when $X \sim F_0$ is $x \mapsto 1-F_0(-x)$. It follows from the calculations above that in the case where the distribution of the signal has its support to the left of some $a_0$, the estimator of the mixing proportion is given by

$$\begin{aligned} \widehat{\alpha }_n = 1 - \inf _{x \le \kappa _0} \frac{1- \mathbb {F}_n(x)}{1-F_b(x)} \end{aligned}$$

where $\kappa _0$ is a known real number satisfying $\kappa _0 \ge a_0$ and $F_b(\kappa _0) < 1$. Furthermore, similar arguments as those used to show Theorem 2.1 yield

$$\begin{aligned} \sqrt{n} (\widehat{\alpha }_n - \alpha _0) \rightarrow _d - \inf _{x \in [a_0, \kappa _0]} \frac{\mathbb {B} \circ (1-F_0)(x)}{1-F_b(x)}. \end{aligned}$$

To estimate $a_0$, the procedure is much similar to the one described for Problem # 1 modulo the mirroring effect. In fact, we have in this case

9 $$\begin{aligned} \widehat{a}_n = \inf \left\{ x \le \kappa _0: 1-\mathbb {F}_n(x) - (1-\widehat{\alpha }_n) (1- F_b(x)) \le \widehat{\alpha }_n \frac{b_n}{\sqrt{n}} \right\} \end{aligned}$$

for a given non-negative sequence $\{b_n\}_{n \ge 1}$ with the same properties as above. The convergence of $\widehat{a}_n$ to $a_0$ in this case can be straightforwardly deduced from the result of Theorem 3.1. For example, if the distribution function of the signal, $F_s$, is differentiable on $(a_0 - \eta , a_0)$ for a small $\eta > 0$ such that the left difference quotient of $F_s$ at the point $a_0$, $f_s(a_0)$, exists and is non-zero, then we can show that

$$\begin{aligned} \frac{\sqrt{n}}{b_n} \Big ( a_0 - \widehat{a}_n \Big ) \overset{\mathbb {P}}{\rightarrow } \frac{1}{f_s(a_0)}. \end{aligned}$$

To decide whether a given $\kappa _0$ satisfies $\kappa _0 \ge a_0$, we can consider $\lambda _0 > \kappa _0$ such that $F_b(\lambda _0) < 1$ and the corresponding statistic

10 $$\begin{aligned} S_n(\kappa _0, \lambda _0):= \sup _{x \in [\kappa _0, \lambda _0]} \sqrt{n} \left| \frac{1-\mathbb { F}_n(x)}{1-F_b(x)} - \frac{1-\mathbb {F}_n(\kappa _0)}{1-F_b(\kappa _0)} \right| . \end{aligned}$$

The null hypothesis $H_0: \kappa _0 \ge a_0$ shall be accepted in case $S_n \le \tilde{s}_{1-\beta }$, where $\tilde{s}_{1-\beta } = \tilde{s}_{1-\beta }(\kappa _0, \lambda _0)$ is the empirical $(1-\beta )$-quantile based on *M* independent copies of the process

$$ \mathcal {S}(\kappa _0,\lambda _0):=\sup _{x \in [\kappa _0, \lambda _0]} \left| \frac{\widetilde{\mathbb {B}}_n \circ \mathbb {F}_n(x)}{1-F_b(x)} - \frac{\widetilde{\mathbb {B}}_n \circ \mathbb {F}_n(\kappa _0)}{1-F_b(\kappa _0)} \right| . $$

Here we used a similar argument as in Section 3.2, together with the fact that under $H_0$, we have $F_s(x) = 1$ for all $x \ge \kappa _0$ and hence $(1-F_0(x))/(1-F_b(x)) = \alpha _0$ for all $x \ge \kappa _0$.

### Estimation of $a_0$ without use of $\kappa _0$

Let us put ourselves again in the setting of Problem #1. If $\kappa _0$ is given, then an estimator of the endpoint $a_0$ is given in Eq. 5. This estimator depends on $\kappa _0$, used in the estimator of the true mixing proportion, $\widehat{\alpha }_n$. See also the expression given in Eq. 4. The goal in this section is to replace $\kappa _0$ by a good “guess” in case it is not available.

Recall that for a given $\kappa _0$, we declare that such a value is appropriate in case we do not find a strong evidence against $H_0$ in the testing problem

$$\begin{aligned} H_0: \kappa _0 \le a_0 \ \ \ \text {versus} \ \ \ \ H_1: \kappa _0 > a_0. \end{aligned}$$

Fixing the asymptotic level at 0.05, this happens in the case where we find that

$$\begin{aligned} T_n(\iota _0, \kappa _0) = \sup _{x \in [\iota _0, \kappa _0] } \sqrt{n} \left| \frac{\mathbb {F}_n(x)}{F_b(x)} - \frac{\mathbb {F}_n(\kappa _0)}{F_b(\kappa _0)} \right| \le \tilde{q}_{0.95}(\iota _0, \kappa _0) \end{aligned}$$

and $\tilde{q}_{0.95}(\iota _0, \kappa _0)$ is the empirical quantile of the statistic $\mathcal {T}_n(\iota _0, \kappa _0)$ defined above in Eq. 8. Given a grid $\mathcal {K}$ of possible values of $\kappa _0$, and a fixed lag $h_0$, we can consider the collection

$$\begin{aligned} \mathcal {T}_n(\kappa - h_0, \kappa ), \ \ \kappa \in \mathcal {K}. \end{aligned}$$

Since the signal density is supported on $(a_0, \infty )$, we can set our estimator for the best $\kappa _0$ to be

11 $$\begin{aligned} \widehat{\kappa }_n:= \max _{\kappa \in \mathcal {K}} \Big \{ T_n(\kappa - h_0, \kappa ) \le \tilde{q}_{0.95}(\kappa -h_0, \kappa ) \Big \}. \end{aligned}$$

For Problem #2, where $f_s$ is known to be supported on $(-\infty , a_0)$, one needs to replace maximization by minimization. More precisely, we compute

12 $$\begin{aligned} \widehat{\kappa }_n:= \min _{\kappa \in \mathcal {K}} \Big \{ T_n(\kappa , \kappa +h_0) \le \tilde{s}_{0.95}(\kappa , \kappa +h_0) \Big \}. \end{aligned}$$

where $\tilde{s}_{0.95}(\kappa , \kappa +h_0)$ is the empirical quantile of the statistic $S_n(\kappa , \kappa +h_0)$ as defined above.

## Numerical Illustration and Data Application

### Settings

In our numerical investigation, we cover 3 shape constraints using different distributions. The settings are shown in Table 1.

**Table 1:** A summary of the settings considered in the paper

Setting	Background distribution	Signal distribution	Constraint	Problem	$a_0$	$\alpha _0$
1a	$\mathcal {U}(0,1)$	$\mathcal {U}(0,0.6)$	Monotone	2	0.6	0.6
1b	$\mathcal {U}(0,1)$	$\mathcal {U}(0,0.6)$	Monotone	2	0.6	0.8
2a	$\mathcal {U}(0,1)$	$0.5 \ \text {Beta}(1,2)/2 + 0.5 \ \mathcal {U}(0,1/2)$	Monotone	2	0.5	0.6
2b	$\mathcal {U}(0,1)$	$0.5 \ \text {Beta}(1,2)/2 + 0.5 \ \mathcal {U}(0,1/2)$	Monotone	2	0.5	0.8
3	$\mathcal {U}(0,1)$	$\text {Beta}(1,2) \cdot 0.7$	Convex	2	0.7	0.8
4	$\mathcal {U}(0,1)$	$\text {Beta}(1,3) \cdot 0.6$	Convex	2	0.6	0.8
5	$\mathcal {N}(0,1)$	$\text {Gamma}(2,1)$	Log-concave	1	0	0.8
6	$\mathcal {N}(0,1)$	$\text {Exp}(1)$	Log-concave	1	0	0.8
7	$\mathcal {N}(0,1)$	$\text {Gamma}(1/2,1)$	Log-concave	1	0	0.8

### Estimation of $a_0$

We start with estimating $a_0$ before exploring consistent estimation of the limit distribution of $\widehat{\alpha }_n$ for the simple reason that the estimator of the latter depends on the estimator of $a_0$. As mentioned above, estimation of $a_0$ can be done using an appropriate $\kappa _0$ or by taking the estimator to be the first/last point at which some test is accepted (see the previous section, in particular Eqs. 11 and 12).

#### Using an Appropriate $\kappa _0$

In Problem #1, we recall that $\kappa _0$ is considered as appropriate if the statistic $T_n(\iota _0, \kappa _0) \le \tilde{q}_{1-\beta }$ for a chosen $\iota _0 < \kappa _0$, where $\tilde{q}_{0.95}$ is an approximation of the 0.95-quantile of the limit distribution of $T_n(\iota _0, \kappa _0)$. In Problem #2, a very similar approach is used, see the statistic $S_n(\kappa _0, \lambda _0)$ defined in Eq. 10. In all the simulations, we have used 1000 replications. To compute the mean, median and standard deviation of $\widehat{a}_n$, we kept only the samples which passed the test (that is the ones which yielded acceptance of $\kappa _0$), computed $\widehat{a}_n$ and selected randomly 900 values thereof. The sample sizes considered are $n =1000$ and 5000. In Table 2 we give the values of $(\iota _0, \kappa _0)$ in the settings of Problem #1 and $(\kappa _0, \lambda _0)$ in those of Problem #2. In all the simulations, we took $b_n = \log (\log (n))$.

**Table 2:** Values of $(\iota _0,\kappa _0)$ in the settings of Problem #1 and values of $(\kappa _0,\lambda _0)$ in the settings of Problem #2

Setting #	$(\kappa _0, \lambda _0)$	$(\iota _0, \kappa _0)$
1a & 2a	(0.8, 0.9)	-
2a & 2b	(0.6, 0.7)	-
3	(0.8, 0.9)	-
4	(0.7, 0.8)	-
5	-	(-1.5, -1)
6	-	(-1.5, -1)
7	-	(-1.5, -1)

**Table 3:** Acceptance rate of $\kappa _0$, the mean, median and standard deviation of $\widehat{a}_n$ (from Eq. 5 in Problem #1 and Eq. 9 in Problem #2) for all the settings of Table 1

Setting #	Acceptance rate		Average		Median		Standard deviation	
	$n=1000$	$n=5000$	$n=1000$	$n=5000$	$n=1000$	$n=5000$	$n=1000$	$n=5000$
1a ($a_0 = 0.6$)	0.942	0.951	0.573	0.587	0.575	0.590	0.009	0.003
1b ($a_0= 0.6$)	0.958	0.954	0.569	0.586	0.570	0.585	0.006	0.002
2a ($a_0 = 0.5$)	0.947	0.938	0.464	0.483	0.465	0.485	0.008	0.003
2b ($a_0= 0.5$)	0.945	0.939	0.457	0.479	0.455	0.480	0.007	0.002
3 ($a_0 = 0.7$)	0.959	0.952	0.537	0.586	0.540	0.585	0.018	0.009
4 ($a_0= 0.6$)	0.951	0.929	0.376	0.422	0.375	0.420	0.017	0.010
5 ($a_0 =0$)	0.949	0.950	0.830	0.571	0.830	0.575	0.0541	0.026
6 ($a_0 =0$)	0.925	0.944	0.046	0.019	0.050	0.020	0.013	0.006
7 ($a_0 =0$)	0.940	0.954	0.033	0.008	0.0350	0.010	0.005	0.002

The number of replications was taken to be 1000. For all simulations, we used $b_n = \log (\log n)$

**Table 4:** Observed probability $P (\widehat{\kappa }_n \in [a_0 - h_0, a_0])$ in Problem #1 and $P (\widehat{\kappa }_n \in [a_0, a_0 + h_0])$ in Problem #2 as well as the mean value and standard deviation of $\widehat{\kappa }_n$ in the settings of Table 1 over 100 replications

Setting #	Probability of $\{\widehat{\kappa }_n \in [a_0-h_0, a_0]\}$			Mean value (sd) of $\widehat{\kappa }_n$		
	or {$\widehat{\kappa }_n \in [a_0, a_0+h_0]\}$					
	500	1000	10000	500	1000	10000
1a ($a_0 = 0.6$)	0.82	0.96	0.97	0.564 (0.110)	0.603 (0.017)	0.602 (0.014)
1b ($a_0 = 0.6$)	0.97	0.97	0.93	0.601 (0.008)	0.602 (0.012)	0.603 (0.012)
2a ($a_0= 0.5$)	0.90	0.91	0.98	0.503 (0.021)	0.507 (0.025)	0.501 (0.007)
2b ($a_0= 0.5$)	0.90	0.93	0.91	0.507 (0.022)	0.505 (0.019)	0.506 (0.020)
3 ($a_0= 0.7$)	0.03	0.07	0.72	0.616 (0.037)	0.648 (0.030)	0.691 (0.025)
4 ($a_0= 0.6$)	0.00	0.01	0.05	0.452 (0.039)	0.483 (0.034)	0.537 (0.026)
5 ($a_0 =0$)	0.02	0.06	0.49	-0.389 (0.461)	-0.358 (0.409)	-0.245 (0.326)
6 ($a_0 =0$)	0.27	0.39	0.54	-0.470 (0.400)	-0.329 (0.370)	-0.252 (0.358)
7 ($a_0 =0$)	0.22	0.39	0.48	-0.430 (0.357)	-0.374 (0.386)	-0.281 (0.354)

The results of Table 3 show on the one hand that the test has the correct level asymptotically (here 0.95), and that the estimator is closer to the true $a_0$ for the larger sample size 5000. On the other hand, they seem to confirm the statement of Theorem 3.1 which says that convergence of $\widehat{a}_n$ should be faster for larger values of $f_s(a_0)$. For example, the convergence of the estimator is quite fast for Settings #1 (a & b), #2 (a & b), #6 and #7 in which $f_s(a_0)$ is either strictly positive or infinite (in Setting #7), and slow for the remaining setting where $f_s(a_0) =0$. Note that there seems to be no significant difference in the performance of the estimator when $\alpha _0 =0.6$ is replaced by $\alpha _0 =0.8$ in Settings #1 and #2.

#### Without use of $\kappa _0$

Since it is not always clear what candidate value of $\kappa _0$ to take, we explore via simulation the performance of the method proposed above for estimating $a_0$ without the use of such a value. In Table 4, we report for sample sizes $n \in \{500,1000,10000\}$ and 100 replications the observed probability of the event $\widehat{\kappa }_n \in [a_0-h_0, a_0]$ in Problem #1 and $\widehat{\kappa }_n \in [a_0, a_0 + h_0]$ in Problem #2, where $h_0$ is the (small) step chosen to construct the grid $\mathcal {K}$. We also report the mean value of $\widehat{\kappa }_n$ in the settings of Table 1. In the Settings #1, #2, #3, and #4 we chose $\mathcal {K} = \{0.2, 0.25, \ldots , 0.75, 0.8\}$, that is $h_0 =0.05$, while in #5, #6, #7, $\mathcal {K} = \{-1, -0.9, \ldots , 0.9, 1\}$, that is $h_0=0.1$.

The results of Table 4 show clearly that this method is very promising for the distributions supported on [0, 1] and when $f_s(a_0) > 0$, which is the case for Settings #1 and #2. In Setting #3, where the distributions are also supported on [0, 1] but $f_s(a_0) =0$, the accuracy of the sample estimator of the probability $P(\widehat{\kappa }_n \in [a_0 - h_0, a_0]$ in Problem #1 and $P(\widehat{\kappa }_n \in [a_0, a_0 + h_0]$ in Problem #2 improves drastically when the sample size is increased from $n=1000$ to 10000. Still in the compactly supported situation, while the performance of the estimate is poor in Setting #4, the average value as well as the standard deviation of $\widehat{\kappa }_n$ show that this estimator can be considered as reasonable. Even tough the signal distribution is both Settings #3 and #4 is a re-scaled Beta distribution, the main reason of the poor performance of $\widehat{\kappa }_n$ in Setting #4, in comparison with Setting #3, is that the density $f_s$ in Setting #4 satisfies $f_s(a_0) = 0$, $f^{(1)}_s(a_0) =0$ and $f^{(2)}_s(a_0)_{-} \ne 0$, where $f^{(j)}_s(a_0)_{-}$ denotes the *j*-th derivative of $f_s$ to the left of $a_0$. Although we do not have a formal proof of this, the results of Theorem 3.1 (re-adapted to Problem #2) suggest that estimation of $a_0$ using $\widehat{\kappa }_n$ should be harder in this case as reflected by the slower convergence rate $(b_n/\sqrt{n})^{1/2} = \sqrt{b_n} n^{-1/4}$ (in comparison with $b_n n^{-1/2}$ in Setting #3 (where $f_s(a_0) =0$ but $f^{(1)}_s(a_0)_{-} \ne 0)$. In Settings #5, #6, #7 the results are also deceiving, especially in Settings #6 and #7. However, while convergence of $\widehat{a}_n$ is very good in these settings with a valid $\kappa _0$ (see again Table 3), we stress again the fact that we lack a general theory for $\widehat{\kappa }_n$ as opposed to the Theorem 3.1 where convergence rates were derived for $\widehat{a}_n$ under different smoothness settings.

##### Remark 2

Although the focus in this section is on estimating $a_0$, it is clear that in the settings where $\widehat{\kappa }_n$ becomes rapidly close to $a_0$ with high probability, this estimator should yield a consistent estimator of the unknown mixing proportion $\alpha _0$. In fact, and if we restrict attention to Problem #1, the estimator $\widehat{\alpha }_n$ given in Eq. 4 should now be replaced by

$$\begin{aligned} \widetilde{\alpha }_n:= 1 - \inf _{x \ge \widehat{\kappa }_n} \frac{\mathbb {F}_(x)}{F_b(x)}. \end{aligned}$$

A close inspection of the arguments used in the proof of Theorem 2.1 (see the supplementary material) reveal that the distribution of $\sqrt{n} (\widetilde{\alpha }_n - \alpha _0)$ is, for *n* large enough, close to that of

$$\begin{aligned} \mathbb {V}_n: = -\inf _{x \in [\widehat{\kappa }_n, a_0]} \frac{\mathbb {B} \circ F_0(x)}{F_b(x)}. \end{aligned}$$

Note that $\widehat{\kappa }_n \in [a_0 - h_0, a_0]$ with high probability. Then,

$$ - \frac{\mathbb {B} \circ F_0(a_0)}{F_b(a_0)} \le \mathbb {V}_n \le - \inf _{x \in [a_0 - h_0, a_0]} \frac{\mathbb {B} \circ F_0(x)}{F_b(x)} $$

and hence $\sqrt{n} (\widetilde{\alpha }_n - \alpha _0) = O_{\mathbb {P}}(1)$. This in turn implies that the estimator $\widetilde{\alpha }_n$ is $n^{-1/2}$-consistent.

### Comparsion with the Approach by Patra and Sen (2016)

In the following, we compare our estimator of the mixing proportion $\widehat{\alpha }_n$ with the one suggested by Patra and Sen (2016). Recall that $F_0$, $F_b$ and $F_s$ are the cumulative distribution functions of the mixture, the background and the signal, respectively. The main approach developed by Patra and Sen (2016) is based on the observation that

$$\begin{aligned} \frac{\mathbb {F}_n - (1-\alpha _0) F_b}{\alpha _0} \end{aligned}$$

is a consistent estimator of the cumulative distribution function of the signal, $F_s$. Since this estimator is not necessarily a distribution function, one can replace it by its isotonic estimator, obtained via a least squares projection on the set of vectors $\{\theta : 0 \le \theta _1 \le \ldots \le \theta _n \le 1\}$. Since $\alpha _0$ is unknown, the authors consider the family of estimators

$$\begin{aligned} \hat{F}^{\gamma }_{s,n} = \frac{\mathbb {F}_n - (1-\gamma ) F_b}{\gamma } \end{aligned}$$

and its isotonic projection $\check{F}^\gamma _{s,n}$ for $\gamma \in (0,1]$. To keep the discussion short, in case $\alpha _0$ is identifiable (which is the case in this paper), then

$$\begin{aligned} \alpha _0 = \inf \left\{ \gamma \in (0,1]: \frac{F_0 - (1-\gamma ) F_b}{\gamma } \ \text {is a CDF} \right\} . \end{aligned}$$

This fact is the basis of the following estimator. Denote by $d^2_n(g, h) = \int (g(x) - h(x))^2 d\mathbb {F}_n(x)$, for any two real functions *g* and *h*. For $\gamma$ close to $\alpha _0$, one expects that $\hat{F}^{\gamma }_{s,b}$ and $\check{F}^{\gamma }_{s,b}$ are close. For some given non-negative sequence $(c_n)_{n \ge 1}$ such that $c_n = o(\sqrt{n})$ and $\lim _{n \rightarrow \infty } c_n = \infty$, define the estimator $\hat{\alpha }^{c_n}_n$ as

$$\begin{aligned} \hat{\alpha }^{c_n}_n:= \inf \left\{ \gamma \in (0,1]: \gamma d_n(\hat{F}^{\gamma }_{s,n}, \check{F}^{\gamma }_{s,n} ) \le \frac{c_n}{\sqrt{n}} \right\} . \end{aligned}$$

Note that $\gamma d_n(\hat{F}^{\gamma }_{s,n}, \check{F}^{\gamma }_{s,n} ) = d_n(\mathbb {F}_n, (1-\gamma ) F_b +\gamma \check{F}^{\gamma }_{s,n} )$. The sequence $(c_n)_{n \ge 1}$, which is acting like a tuning parameter, is crucial for the good performance of the estimation method. Patra and Sen (2016) investigated a 10-fold cross-validation procedure to make the best selection among some given choices. However, and as the authors themselves note, this technique can be numerically very expensive. Another recommendation they made in their paper and on which they base many simulation results is to take

$$\begin{aligned} c_n = 0.1 \log (\log n). \end{aligned}$$

From their Theorem 4, it follows that if $c_n = o(n^{1/4})$, then

$$ \frac{\sqrt{n}}{c_n} (\hat{\alpha }^{c_n}_n - \alpha _0) \overset{\mathbb {P}}{\rightarrow } c , $$

where $c < 0$ depends on $F_0$, $F_b$ and $\alpha _0$. Thus, the convergence rate of $\hat{\alpha }^{c_n}_n$ is a bit slower than our parametric rate for *n* large enough.

**Table 5:** The mean squared errors as well as the mean values (in brackets), obtained over 100 replications, for our estimator and the estimators of Patra and Sen (2016), $\widehat{\alpha }^{c_n}_n$ with $c_n = 0.1 \log (\log n)$ and $\widehat{\alpha }^{cv}_n$ in all the settings considered in this paper

Setting #	$n=100$			$n=500$			$n=1000$		
	$\widehat{\alpha }_n$	$\hat{\alpha }^{c_n}_n$	$\hat{\alpha }^{cv}_n$	$\widehat{\alpha }_n$	$\hat{\alpha }^{c_n}_n$	$\hat{\alpha }^{cv}_n$	$\widehat{\alpha }_n$	$\hat{\alpha }^{c_n}_n$	$\hat{\alpha }^{cv}_n$
1a ($\alpha _0 =0.6$)	0.098	0.127	0.170	0.047	0.084	0.074	0.030	0.065	0.054
	(0.644)	(0.497)	(0.648)	(0.616)	(0.526)	(0.615)	(0.609)	(0.540)	(0.610)
1b ($\alpha _0 =0.8$)	0.064	0.158	0.111	0.032	0.098	0.051	0.024	0.076	0.041
	(0.818)	(0.649)	(0.783)	(0.813)	(0.704)	(0.812)	(0.809)	(0.725)	(0.810)
2a ($\alpha _0 =0.6$)	0.082	0.106	0.149	0.038	0.061	0.087	0.026	0.0507	0.064
	(0.625)	(0.524)	(0.653)	(0.614)	(0.549)	(0.629)	(0.607)	(0.556)	(0.615)
2b ($\alpha _0 =0.8$)	0.064	0.123	0.104	0.031	0.075	0.057	0.020	0.057	0.031
	(0.818)	(0.689)	(0.801)	(0.810)	(0.729)	(0.816)	(0.808)	(0.745)	(0.807)
3 ($\alpha _0 =0.8$)	0.090	0.144	0.110	0.044	0.097	0.064	0.032	0.084	0.040
	(0.855)	( 0.665)	(0.798)	(0.827)	(0.706)	( 0.820)	(0.816)	(0.718)	(0.810)
4 ($\alpha _0 =0.8$)	0.080	0.109	0.091	0.036	0.073	0.049	0.025	0.055	0.034
	(0.848)	(0.702)	(0.806)	(0.819)	(0.731)	(0.803)	(0.814)	(0.746)	(0.802)
5 ($\alpha _0 =0.8$)	0.098	0.115	0.179	0.046	0.068	0.065	0.034	0.051	0.032
	(0.872)	(0.695)	(0.626)	(0.831)	(0.735)	(0.743)	(0.825)	(0.751)	(0.774)
6 ($\alpha _0 =0.8$)	0.095	0.148	0.232	0.044	0.082	0.071	0.034	0.061	0.036
	(0.862)	(0.661)	(0.572)	(0.828)	(0.721)	(0.740)	(0.822)	(0.742)	(0.773)
7 ($\alpha _0 =0.8$)	0.092	0.157	0.248	0.047	0.081	0.065	0.031	0.063	0.040
	(0.855)	(0.653)	(0.557)	(0.829)	(0.723)	(0.743)	(0.819)	(0.739)	(0.773)

To draw a comparison between our approach and the one by Patra and Sen (2016), we compute their estimator with the fixed sequence $c_n = 0.1 \log (\log n)$ and also the one obtained using the 10-fold cross-validation, in addition to our estimator. The estimators are computed for all the settings considered in this paper. Based on 100 replications, we report in Table 5 the mean squared errors as well as the average value of each of the estimators. In computing our estimator, we used the same values of $\kappa _0$ as above. The results show that in almost all of the settings, our estimator has the smallest mean squared error. Moreover, it is much faster to compute than $\hat{\alpha }^{c_n}_n$. The slow computation of $\hat{\alpha }^{c_n}_n$ is due to the isotonization step which we do not have in our approach. The estimator obtained with cross-validation takes a prohibitive computational time for sample sizes of 10000, and this is the reason we restricted attention in Table 5 to much smaller sample sizes. Thus, the rapid computation and fast rate of convergence are appealing advantages of our approach. However, we need to point out that the value of $\kappa _0$ needs to be judiciously chosen, otherwise the obtained estimator will be erroneous. We refer the reader to Section 3.2, where a test of whether $\kappa _0$ is appropriate is constructed.

### Approximation of the Limit Distribution of $\widehat{\alpha }_n$

Our aim in this section is to validate Theorem 2.1. The convergence in distribution given in that theorem is for Problem #1. We recall here these limit convergences for both problems: If $\mathbb {B}$ is a standard Brownian Bridge from (0, 0) to (1, 0), then

- Problem #1: For any $\kappa _0 \le a_0$ such that $F_b(\kappa _0) > 0$,
  $$\begin{aligned} \sqrt{n} (\hat{\alpha }_n - \alpha _0) \rightarrow _d -\inf _{x \in [\kappa _0, a_0]} \frac{\mathbb {B} \circ F_0(x)}{F_b(x)}. \end{aligned}$$
- Problem #2: For any $\kappa _0 \ge a_0$ such that $F_b(\kappa _0) < 1$,
  $$\begin{aligned} \sqrt{n} (\widehat{\alpha }_n - \alpha _0) \rightarrow _d - \inf _{x \in [a_0, \kappa _0]} \frac{\mathbb {B} \circ (1-F_0)(x)}{1-F_b(x)}. \end{aligned}$$

The limit distribution of $\widehat{\alpha }_n$ can be very well approximated through generating a large number of independent processes which are very close to $\mathbb {B}$. This can be done by generating i.i.d. uniform random variables $U_1, \ldots , U_N \in [0,1]$ for a large number *N*. Let $\mathbb {G}_N$ denote the empirical distribution function of $U_1, \ldots , U_N$, that is, $\mathbb {G}_N(t) = N^{-1} \sum _{j=1}^N \mathbb {I}_{\{U_j \le t \}}, t \in \mathbb {R}$. Then, the distributions

$$\begin{aligned} - \inf _{x \in [\kappa _0, a_0]} \frac{\sqrt{N} \big \{\mathbb {G}_N \circ F_0(x) - F_0(x) \big \} }{F_b(x)} \end{aligned}$$

and

$$\begin{aligned} - \inf _{x \in [a_0, \kappa _0]} \frac{\sqrt{N} \big \{\mathbb {G}_N \circ (1-F_0(x)) - (1- F_0(x)) \big \} }{1-F_b(x)} \end{aligned}$$

are good approximations for the distributions of the limit random variables above.

**Fig. 1:**
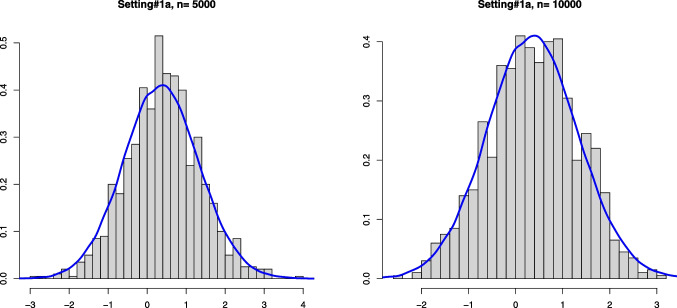
Histograms of the estimation error associated with $\widehat{\alpha }_n$ for Setting #1a ($\alpha _0 =0.6$, $a_0=0.6$ and we take $\kappa _0=0.7$) and the kernel density estimator of the limit distribution (solid line)

**Fig. 2:**
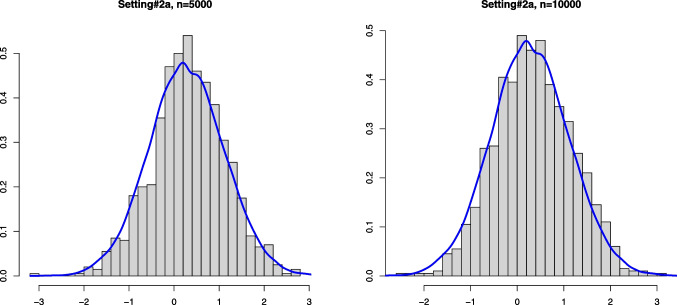
Histograms of the estimation error associated with $\widehat{\alpha }_n$ for Setting #2a ($\alpha _0 =0.6$, $a_0=0.5$ and we take $\kappa _0=0.6$) and the kernel density estimator of the limit distribution (solid line)

**Fig. 3:**
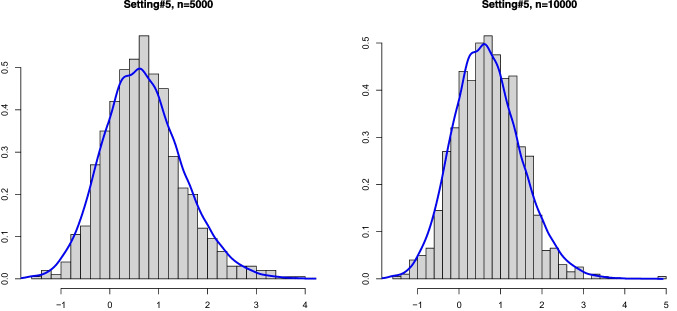
Histograms of the estimation error associated with $\widehat{\alpha }_n$ for Setting #5 ($\alpha _0 =0.8$, $a_0=0$ and we take $\kappa _0=-1$) and the kernel density estimator of the limit distribution (solid line)

**Fig. 4:**
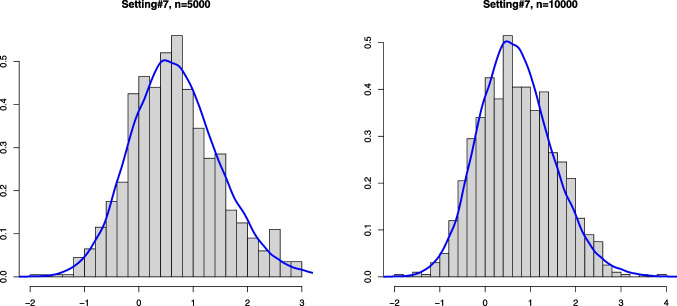
Histograms of the estimation error associated with $\widehat{\alpha }_n$ for Setting #7 ($\alpha _0 =0.8$, $a_0=0$ and we take $\kappa _0=-1$) and the kernel density estimator of the limit distribution (solid line)

On the other hand, drawing from the same mixture model allows to obtain several realizations of the scaled estimation error $\sqrt{n}(\widehat{\alpha }_n - \alpha _0)$. The plots below show the histogram of 1000 such realizations in the Settings #1, #2, #5 and #7. Also, based on $N = 50000$, we constructed a kernel estimator of the density of the limit distribution in each of the settings. These kernel density estimators should be seen as the true densities to which the histograms for the sample sizes $n=5000$ and $n=10000$ are compared. The simulation results are quite conform with the theory; see Figs. 1, 2, 3 and 4.

### Non-parametric Estimation of the Signal Density under Shape Constraints

#### Estimation of the Signal Density under Monotonicity and Convexity

In this section, we put ourselves in the setting of Langaas et al. (2005) in which the mixture distribution is compactly supported on [0, 1] with $F_b$ being the uniform distribution on this interval and $F_s$ admits a density $f_s$ with respect to Lebesgue measure. Thus, the true mixture density is given by

$$\begin{aligned} f_0(x) = 1-\alpha _0 + \alpha _0 f_s(x), \ \ x \in [0,1]. \end{aligned}$$

Note that our $\alpha _0$ is equal to $1-\pi _0$ in the notation used by Langaas et al. (2005). In their work, the authors assume that $f_s(1) =0$, a condition that guarantees identifiability. Furthermore, the authors consider the following shape-constrained conditions on $f_s$:

- decreasing on [0, 1],
- convex and decreasing on [0, 1].

These assumptions are used with the main goal of coming up with a good estimator of $\alpha _0$. Under the monotonicity assumption, the authors consider two estimators which are based on the Grenander estimator of $f_0$; see their Sections 4.2 and 4.3. Under the additional convexity assumption, their estimator is equal to the value of the convex and decreasing MLE of $f_0$ of Groeneboom et al. (2001) at the point 1.

In this section, we shall combine the shape constraints above with the additional assumption that the support of $f_s$ is $[0, a_0]$ for some $a_0 < 1$. This assumption implies $f_s(1)=0$ made by the authors and therefore is stronger. However, in the context of multiple testing, it is easily conceivable that the density $f_s$, which is also the density under the alternative, admits a support that is strictly included in [0, 1]. Given $\kappa _0 \ge a_0$ such that $\kappa _0 < 1$, recall that our estimators of $\alpha _0$ and $a_0$ are respectively given by

$$\begin{aligned} \widehat{\alpha }_n = 1 - \inf _{x \in [0, \kappa _0]} \frac{1- \mathbb {F}_n(x)}{1-x} \end{aligned}$$

and

$$\begin{aligned} \widehat{a}_n = \inf \left\{ x \in [0,\kappa _0]: 1-\mathbb {F}_n(x) - (1-\widehat{\alpha }_n) (1- x) \le \widehat{\alpha }_n \frac{b_n}{\sqrt{n}} \right\} \end{aligned}$$

for an appropriately chosen non-negative sequence $\{b_n\}_{n \ge 1}$. Note that $\widehat{a}_n \le a_0$ with probability tending to 1 as $n \rightarrow \infty$. This can be shown using the same argument as in the proof of Theorem 3.1.

##### Monotonicity

We start in this section with the case where $f_s$ is non-increasing on [0, 1] and supported on $[0, a_0]$ with $a_0 < 1$. Then, $f_0$ is non-increasing on [0, 1], and has a plateau at the value $1-\alpha _0$ on the subinterval $[a_0,1]$. Consider $\widehat{f}_n$ the Grenander estimator of $f_0$, which is equal to the left first derivative of the least concave majorant (LCM) of the empirical distribution function

$$\begin{aligned} \mathbb {F}_n(t) = n^{-1} \sum _{i=1}^n \mathbb {I}_{\{ X_i \le t\}}, \ \ t \in \mathbb {R}. \end{aligned}$$

A natural estimator of the signal density $f_s$ is

13 $$\begin{aligned} \widehat{f}_{n, s} = \frac{\widehat{f}_n - 1 + \widehat{\alpha }_n}{\widehat{\alpha }_n} \vee 0 \end{aligned}$$

which is non-increasing on [0, 1]. The asymptotic theory for the Grenander estimator under specific regularity conditions is well established. Let $x_0 \in [0, a_0)$. We will distinguish between two cases.

##### Case 1

Here, it is assumed that $f_s$ is continuously differentiable in a small neighborhood of $x_0$ with $f'_s(x_0) < 0$. Then, it follows from Grenander (1956) and the fact that $\widehat{\alpha }_n$ is $\sqrt{n}$-consistent that

14 $$\begin{aligned} n^{1/3} \left( \widehat{f}_{n, s}(x_0) - f_s(x_0) \right) \rightarrow _d \frac{1}{\alpha _0} \left| 4 f_0(x_0) f'_0(x_0) \right| ^{1/3} \mathbb {C} \end{aligned}$$

where $\mathbb {C} \overset{d}{=}\ \text {argmax}_{t \in \mathbb {R}} \{ \mathbb {W}(t) - t^2 \}$ with $\mathbb {W}$ a two-sided Brownian motion originating from 0. The distribution of $\mathbb {C}$ is well-known under the name of *Chernoff’s distribution*; see e.g., Chernoff (1964) and Groeneboom and Wellner (2001). In case $f_s$ is strictly decreasing and continuously differentiable on $(0, a_0)$, then the weak convergence in Eq. 14 is valid for any $x_0 < \widehat{a}_n$ with probability tending to 1. This is an immediate consequence of Eq. 13 and the fact that we have $x_0 < a_0$ in this case. Furthermore, the same convergence can be combined with consistent estimation of $f'_0(x_0)$ to obtain confidence intervals for $f_s(x_0)$ with the correct asymptotic level. To estimate $f'_0(x_0)$ one can for example resort to using a kernel estimator. However, we prefer to make use of the Grenander estimator to this aim. More precisely, in case the true density $f_s$ is continuously differentiable in a small neighborhood of $x_0$ such that $f'_0(x_0) < 0$, then it follows from Theorem 2.1 of Durot et al. (2012) that

15 $$\begin{aligned} \sup _{ t \in I_n(x_0)} \vert \widehat{f}_n(t) - f_0(t) \vert = O_{\mathbb {P}} \left( \frac{\log n}{n}\right) ^{1/3} \end{aligned}$$

where

$$\begin{aligned} I_n(x_0) = \left[ x_0 - c \frac{(\log n)^{2/3}}{n^{1/3}}, x_0 + c \frac{(\log n)^{2/3}}{n^{1/3}}\right] \end{aligned}$$

for some $c > 0$. Put $\delta _n = c (\log n)^{2/3} n^{-1/3}$. It follows that

$$\begin{aligned} \widehat{f}_{n}(x_0 \pm \delta _n)= &  f_0(x_0 \pm \delta _n) + O_{\mathbb {P}} \left( \frac{\log n}{n}\right) ^{1/3} \\= &  f_0(x_0) \pm \delta _n f'_0(x_0) + o(\delta _n) + O_{\mathbb {P}} \left( \frac{\log n}{n}\right) ^{1/3} \end{aligned}$$

using Taylor expansion up to order 1. Therefore,

$$\begin{aligned} \widehat{f}_{n}(x_0 + \delta _n) - \widehat{f}_{n}(x_0 - \delta _n) = 2 \delta _n f'_0(x_0) + o(\delta _n) + O_{\mathbb {P}} \left( \frac{\log n}{n}\right) ^{1/3}. \end{aligned}$$

Since $(\log n/n)^{1/3} = o(\delta _n)$ it follows that

$$\begin{aligned} \widehat{d}_n(x_0): = \frac{\widehat{f}_{n}(x_0 + c n^{-1/3} (\log n)^{2/3}) - \widehat{f}_{n}(x_0 - c n^{-1/3} (\log n)^{2/3})}{2 c n^{-1/3} (\log n)^{2/3}} \end{aligned}$$

is a consistent estimator of $f'_0(x_0)$.

The quantiles of the distribution of $\mathbb {C}$ can be obtained by generating a large number $B > 0$ of drifted Brownian motions $\mathbb {W}_b$ for which one has to determine the location of the maximum of $t \mapsto \mathbb {W}_b(t) -t^2$ on some large interval. Alternatively, a very good approximation of $\mathbb {C}$ can be obtained by computing the Grenander estimator of a known monotone density (for some large sample). A very accurate approximation of several quantiles of $\mathbb {C}$ was obtained and published in Table 1 in Groeneboom and Wellner (2001). The approximation in that paper is based on solving the partial differential (heat) equation satisfied by the Chernoff density and the thorough analysis of this equation done in Groeneboom (1989). In Table 6 we gather some of these quantiles which were very kindly sent to us by Piet Groeneboom. An asymptotic confidence interval of $f_s(x_0)$ of level 0.95 can be taken to be

16 $$\begin{aligned} \Big [ \widehat{f}_{n, s}(x_0) - 0.998 n^{-1/3} \frac{1}{\widehat{\alpha }_n} \left| 4 \widehat{f}_n(x_0) \widehat{d}_n(x_0) \right| ^{1/3}, \nonumber \\ \widehat{f}_{n, s}(x_0) + 0.998 n^{-1/3} \frac{1}{\widehat{\alpha }_n} \left| 4 \widehat{f}_n(x_0) \widehat{d}_n(x_0) \right| ^{1/3} \Big ]. \end{aligned}$$

**Table 6:** Accurate approximation of some quantiles of the Chernoff distribution

$\alpha$	Quantile
0.900	0.6642351969
0.950	0.8450811902
0.975	0.9981810993
0.990	1.1715343420
0.995	1.2866587967
0.999	1.5166635630

##### Case 2

In this second case, we consider the situation where the signal density $f_s$ has a flat part and that $x_0$ belongs to its interior. Let [*a*, *b*] denote the closure of the largest interval containing $x_0$ and over which $f_s$ is constant. Since $f_0$ is a mixture of a uniform density and $f_s$, [*a*, *b*] plays a similar role for $f_0$. As established in Carolan and Dykstra (1999), it holds that

17 $$\begin{aligned} n^{1/2} \left( \frac{\widehat{f}_{n}(x_0)}{ f_0(x_0)} - 1\right) \rightarrow _d \sqrt{\frac{1-p}{p}} Z + \frac{1}{\sqrt{p}} \mathbb {D} \end{aligned}$$

where $p = (b-a) f_0(x_0) = F_0(b) - F_0(a)$, $Z \in \mathcal {N}(0,1)$ and $\mathbb {D}$ is the left derivative at $(x_0 - a)/(b-a)$ of the least concave majorant of a standard Brownian Bridge $\mathbb {B}$ that is independent of *Z*. With probability tending to 1, we have that

$$\begin{aligned} \widehat{f}_{n, s}(x_0) = \frac{\widehat{f}_n(x_0) - 1 + \widehat{\alpha }_n}{\widehat{\alpha }_n} \end{aligned}$$

and hence

$$\begin{aligned} \frac{\widehat{f}_{n}(x_0)}{ f_0(x_0)} -1= &  \frac{1 }{f_0(x_0)} \left( \widehat{\alpha }_n \widehat{f}_{n, s}(x_0) - \widehat{\alpha }_n +1 - (1-\alpha _0) - \alpha _0 f_s(x_0) \right) \\= &  \frac{1 }{f_0(x_0)} \left( \widehat{\alpha }_n \widehat{f}_{n, s}(x_0) -\alpha _0 f_s(x_0) - \widehat{\alpha }_n + \alpha _0 \right) \\= &  \frac{1 }{f_0(x_0)} \left( (\widehat{\alpha }_n - \alpha _0) \widehat{f}_{n, s}(x_0) + \alpha _0 (\widehat{f}_{n, s}(x_0) - f_s(x_0)) - \widehat{\alpha }_n + \alpha _0 \right) \end{aligned}$$

implying that

$$\begin{aligned} \widehat{f}_{n, s}(x_0) - f_s(x_0) = \frac{f_0(x_0)}{\alpha _0} \left( \frac{\widehat{f}_{n}(x_0)}{ f_0(x_0)} -1 \right) + \frac{1}{\alpha _0}(\widehat{\alpha }_n - \alpha _0) (1- \widehat{f}_{n, s}(x_0)). \end{aligned}$$

The identity above, the weak convergence in Eq. 17 and the result of Theorem 2.1 yield

$$\begin{aligned} n^{1/2} \left( \widehat{f}_{n, s}(x_0) - f_s(x_0) \right) \rightarrow _d \mathbb {L} \end{aligned}$$

where $\mathbb {L}$ is a random variable whose distribution depends on the joint stochastic behavior of the estimators $\widehat{\alpha }_n$ and $\widehat{f}_n(x_0)$. While determining the exact distribution of $\mathbb {L}$ is out of the scope of this paper, we can resort to using bootstrap in order to obtain asymptotic confidence intervals. As shown in Sen et al. (2010), bootstrapping samples of size *n* from the empirical distribution or the Grenander estimator does not yield consistent estimators. One way to solve this issue is to resort to *m* out of *n* bootstrap. This means that the bootstrapped samples should be of size $m = m_n = o(n)$. Consistency of this kind of bootstrap was shown for a non-increasing density which admits a strictly negative derivative at $x_0$. However, our simulations do not seem to support this result. In fact, the confidence intervals we obtained for estimating $f_s(x_0)$ in Setting #2 had systematically a significantly lower coverage than the targeted asymptotic level. This is the reason for which we resort to another way of constructing such intervals (see below). However, we conjecture that the *m* out of *n* bootstrap from the Grenander estimator is consistent when the monotone density is flat. More precisely, let $g_0$ be a non-increasing density which is flat and $X_1, \ldots , X_n \overset{i.i.d.}{\sim }\ g_0$. Also, let $\hat{g}_n$ denote the Grenander estimator of $g_0$ based on $X_1, \ldots , X_n$, and let $\hat{g}^*_n$ denote the Grenander estimator based on $X^*_1, \ldots , X^*_{m_n} \overset{i.i.d.}{\sim }\ \hat{g}_n$ . If

$$\begin{aligned} \Delta _n = \sqrt{n} (\hat{g}_n(x_0) - g_0(x_0)),\ \text {and} \ \ \Delta ^*_n = \sqrt{m_n} (\hat{g}^*_n(x_0) - \hat{g}_n(x_0)) \end{aligned}$$

then we conjecture that $\lim _{n \rightarrow \infty } \sup _{x \in \mathbb {R}} \left| P(\Delta _n \le x) - P(\Delta ^*_n \le x) \right| =0$.

In this approach, *B* samples are drawn from the obtained Grenander estimator, and using each of these samples, a new Grenander estimator $\hat{f}^*_n$ is computed as well as the monotone estimator of $f_s$, $\hat{f}^*_{n, s}$, according to Eq. 13. To draw samples from the original Grenander estimator, let us recall that this estimator is given by

$$\begin{aligned} c_1 \mathbb {I}_{[0, \tau _1)} + c_2 \mathbb {I}_{[\tau _1, \tau _2)} + \ldots + c_p \mathbb {I}_{[\tau _{p-1}, \tau _p)} \end{aligned}$$

where $\tau _1< \ldots < \tau _p$ are the knot points and $c_1, \ldots , c_p$ are the positive values taken by the estimator on each “block”. Thus, the estimator can be regarded as a mixture density with weights $w_i = c_i (\tau _i - \tau _{i-1}), i =1, \ldots , p$ (with $\tau _0 =0$) and uniform density components. One can first generate a multinomial vector with probabilities $(w_1, \ldots , w_p)$. If the *k*-th component of this vector is 1, then one generates a number from the uniform density on $[\tau _{k-1}, \tau _k]$. Each bootstrap sample yields a new estimate of $\alpha _0$ and this estimate is then used to construct the bootstrap Grenander estimator for the signal density $f_s$. The *B* obtained estimators can be then used to obtain lower and upper quantiles $\sqrt{m_n} (\hat{f}^*_{n, s}(x_0) - \hat{f}_{n, s}(x_0))$. If $q^*_{\alpha /2}$ and $q^*_{1-\alpha /2}$ denote these quantiles, then we take the asymptotic confidence interval for $f_s(x_0)$ to be

18 $$\begin{aligned} \left[ \hat{f}_{n,s}(x_0) - \frac{q^*_{1-\alpha /2}}{\sqrt{n}}, \hat{f}_{n,s}(x_0) - \frac{q^*_{\alpha /2}}{\sqrt{n}} \right] . \end{aligned}$$

We give in the following table the coverage of the confidence interval for $f_s(x_0)$ for $x_0 \in \{0.3, 0.4\}$ in Setting #1 and $x_0 \in \{0.2, 0.3\}$ in Setting #2. In the latter setting, we also give the coverage of the confidence interval in Eq. 16. In fact, we have in this case that

$$\begin{aligned} f_s(x) = \frac{1}{2} \left( 4(1-2x) + 2 \right) \mathbb {I}_{\{x \in [0,1/2]\}} = (3-4x) \mathbb {I}_{\{x \in [0,1/2]\}} \end{aligned}$$

with $$f'_s(x) = -4 $$ implying that $$f'_0(x) = - 4 \alpha _0$$ for all $$x \in (0,1/2)$$.

**Fig. 5:**
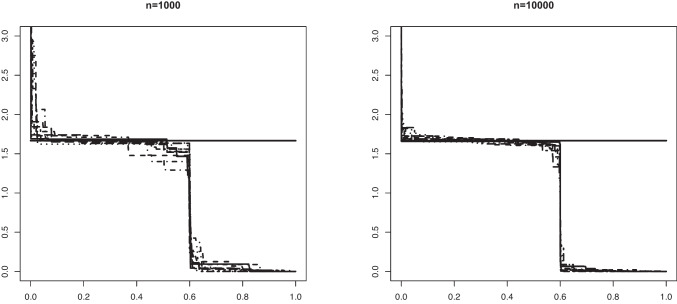
An example in Setting #1a of the Grenander estimator of the uniform density $f_s(x) = 5/3 \mathbb {I}_{[0, 0.6]}$. The figure shows 10 such estimators based on independent samples of size $n=1000$ (left) and $n=10000$ (right). The mixing proportion is $\alpha _0 = 0.6$. The solid horizontal line depicts the value 5/3 that the estimators aim to learn

**Fig. 6:**
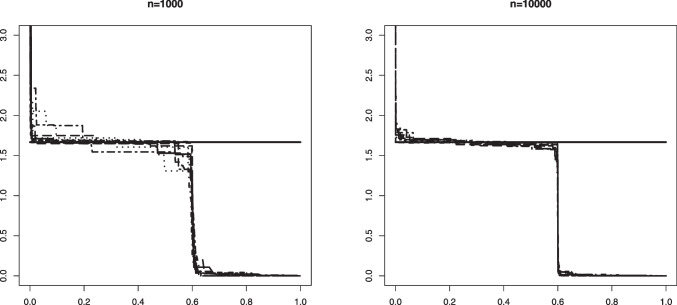
An example in Setting #1b of the Grenander estimator of the uniform density $f_0(x) = 5/3 \mathbb {I}_{[0, 0.6]}$. The figure shows 10 such estimators based on independent samples of size $n=1000$ (left) and $n=10000$ (right). The mixing proportion is $\alpha _0 = 0.8$. The solid horizontal line depicts the value 5/3 that the estimators aim to learn

**Fig. 7:**
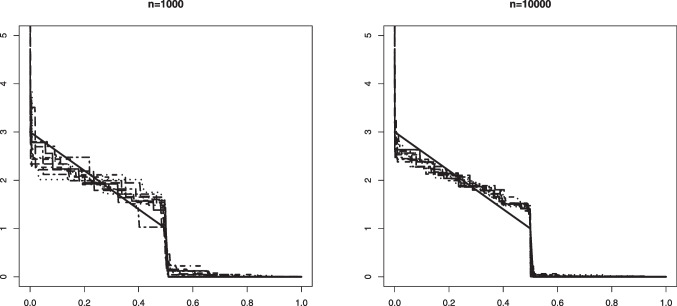
An example in Setting #2a of the Grenander estimator of the density $f_s(x) = (3-4x) \mathbb {I}_{[0, 0.5]}$ (depicted by the solid line). The figure shows 10 such estimators based on independent samples of size $n=1000$ (left) and $n=10000$ (right). The mixing proportion is $\alpha _0 = 0.6$

**Fig. 8:**
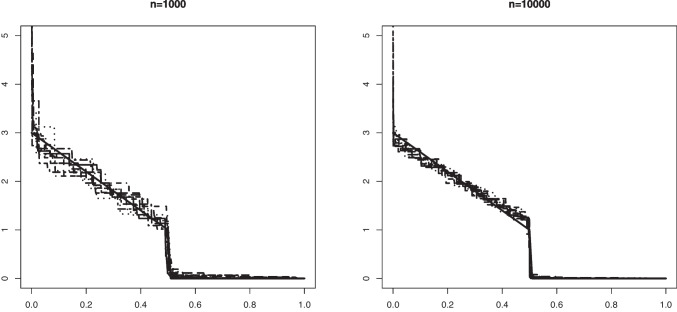
An example in Setting #2b of the Grenander estimator of the density $f_s(x) = (3-4x) \mathbb {I}_{[0, 0.5]}$ (depicted by the solid line). The figure shows 10 such estimators based on independent samples of size $n=1000$ (left) and $n=10000$ (right). The mixing proportion is $\alpha _0 = 0.8$

In Figs. 5 and 6 we plotted 10 independent Grenander estimators of the uniform density on [0, 0.6] in Settings #1a and #1b. Convergence to the constant 5/3 is clearly seen for the larger sample size $n=10000$. We also note a better performance in the latter setting with the larger value of $\alpha _0 =0.8$, which is to be expected. The Grenander estimators shown in Figs. 7 and 8 for the Settings #1b and #2b show similar features. The main difference to be noted is that the estimators have more kink points in this case since the first derivative of the true density is not strictly negative on its support.

The empirical coverage of the 95% asymptotic confidence intervals described above is reported in Table 7 (for Settings #1a and #1b) and Table 8 (for Settings #2a and #2b) for some chosen points $x_0$. For the former setting, we took $m =m_n= \lfloor n^{2/3} \rfloor$ in the *m* out of *n* bootstrap and for the latter $c=0.5$ in estimating the derivative of the true mixed density. We note that the obtained coverages are quite close to the nominal one.

**Table 7:** Empirical coverage of the asymptotic confidence interval of $f_s(x_0)$ in Eq. 18 for the Settings # 1a and #1b based on $m_n$ out of *n* bootstrapping from the Grenander estimator with $m_n = \lfloor n^{2/3} \rfloor$

$$x_0 $$	Setting #1a			Setting #1b		
	$n=1000$	$n=5000$	$n=10000$	$n=1000$	$n=5000$	$n=10000$
0.3	93	89	97	92	97	93
0.4	96	89	97	96	93	94

The number of replications was taken to be $M=100$ while the quantiles of the bootstrapped distribution were estimated using $B=500$ bootstrap samples

**Table 8:** Empirical coverage of the asymptotic confidence interval of $f_s(x_0)$ as given in Eq. 18 for the Settings #2a and #2b

$$x_0 $$	Setting #2a			Setting #2b		
	$n=1000$	$n=5000$	$n=10000$	$n=1000$	$n=5000$	$n=10000$
0.2	92	92	86	91	97	85
0.3	98	90	87	98	98	91

In estimating the derivative $f'_0(x_0)$ we took $c= 0.5$. Also, the number of replications was chosen to be $M=100$

**Table 9:** Estimates of some quantiles of $\mathbb {H}''(0)$

$\alpha$	Quantile
0.025	-2.437220
0.05	-2.016842
0.10	-1.572602
0.900	1.585226
0.950	1.977245
0.975	2.336658
0.990	2.591252
0.995	2.736833
0.999	3.090453

See the main text for more details

##### Monotonicity and convexity

In this section, we assume that $$f_s$$ is convex and non-increasing on [0, 1] and supported on $$[0,a_0] $$. Then, $$f_0$$ is convex and non-increasing on [0, 1] and hence can be estimated with the convex LSE or MLE constructed and studied in Groeneboom et al. (2001). Call one of these estimators $$\widehat{f}_n$$. Recall that the estimator of the signal density is given by

$$\begin{aligned} \widehat{f}_{n,s} = \frac{\widehat{f}_n - 1 + \widehat{\alpha }_n}{\widehat{\alpha }_n} \vee 0. \end{aligned}$$

As in the monotone case, two cases can be distinguished:

##### Case 1

We assume that $f_0$ (or equivalently $f_s$) is twice continuously differentiable in the neighborhood of $x_0$ such that $f''_0(x_0) < 0$ (or equivalently $f''_s(x_0) < 0$). Then, as $n \rightarrow \infty$, the following joint convergence in distribution holds:

$$\begin{aligned} \left( \begin{array}{ll} n^{2/5} \left( \widehat{f}_{n, s}(x_0) - f_s(x_0) \right) \\ n^{1/5} \left( \widehat{f}'_{n, s}(x_0) - f'_s(x_0) \right) \end{array} \right) \rightarrow _d \frac{1}{\alpha _0} \left( \begin{array}{ll} \left[ \frac{f^2_0(x_0) f''_0(x_0)}{24}\right] ^{1/5} \ \ \mathbb {H}''(0) \\ \left[ \frac{f_0(x_0) (f''_0(x_0))^3}{24^3}\right] ^{1/5} \mathbb {H}^{(3)}(0) \end{array} \right) \end{aligned}$$

where $\mathbb {H}$ is the a.s. convex envelope of the drifted Gaussian process $\{\mathbb {Y}(t) = \int _0^{t} \mathbb {W}(s) ds + t^4, t \in \mathbb {R} \}.$ This joint weak convergence is very hard to derive and the main result of Groeneboom et al. (2001). The distribution of $\mathbb {H}''(0)$ and its quantiles can be approximated by computing the convex MLE or LSE of a known convex density at some fixed point. Such a known density should be chosen so that it satisfies the required regularity assumptions. For example, one can take the density of a Beta distribution with shape parameters 1 and 3 given by

$$\begin{aligned} g_0(x) = 3 (1-x)^{2} \mathbb {I}_{x \in [0,1]}. \end{aligned}$$

If $\widetilde{g}_N$ is the convex MLE or LSE based on a random sample of size *N*, then with $x_0 = 1/2$ and as $N \rightarrow \infty$ we have that

$$\begin{aligned} \left( \frac{64}{9}\right) ^{1/5} N^{2/5} \left( \widetilde{g}_N(1/2) - \frac{3}{4} \right) \rightarrow _d \mathbb {H}''(0). \end{aligned}$$

Both convex estimators can be computed using a support reduction algorithm; see e.g. Groeneboom et al. (2008). The quantile values presented in Table 9 are obtained by computing $M=1000$ replications of the convex LSE of the Beta density $g_0$ above for $N=10^5$.

**Fig. 9:**
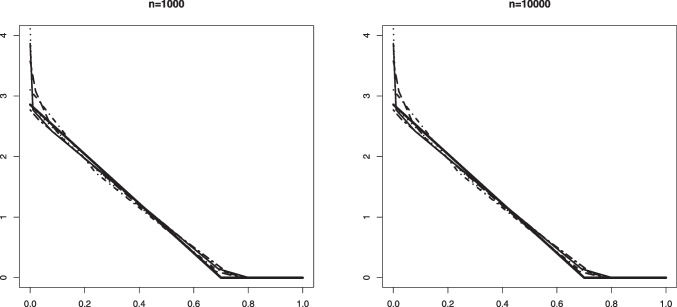
An example in Setting #3 of the convex LSE of the density $f_s(x) = 2/0.7 (1-x/0.7) \mathbb {I}_{x \in [0,0.7]}$ (depicted by the solid line). The convex LSE was computed using a support reduction algorithm. The figure shows 5 such estimators based on independent samples of size $n=1000$ (left) and $n=10000$ (right). The mixing proportion is $\alpha _0 = 0.8$

**Fig. 10:**
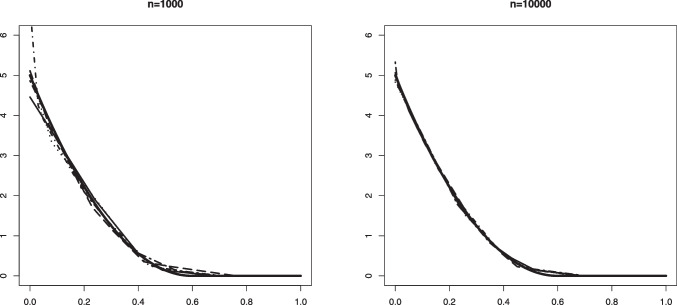
An example in Setting #4 of the convex LSE of the density $f_s(x) = 3/0.6 (1-x/0.6)^2 \mathbb {I}_{x \in [0,0.6]}$ (depicted by the solid line). The convex LSE was computed using a support reduction algorithm. The figure shows 5 such estimators based on independent samples of size $n=1000$ (left) and $n=10000$ (right). The mixing proportion is $\alpha _0 = 0.8$

From now on, $\widehat{f}_n$ denotes the convex LSE as defined in Groeneboom et al. (2001). To construct confidence intervals in this case, we need to compute an estimator of $f''_0(x_0)$. Analog to the monotone case, we can take the following estimator

$$\begin{aligned} \widehat{d}_n(x_0) = \frac{\widehat{f}_n(x_0 +\delta _n) + \widehat{f}_n(x_0 - \delta _n) - 2 \widehat{f}_n(x_0)}{\delta ^2_n} \end{aligned}$$

with $\delta _n = c (\log n)^{2/5} n^{-1/5}$. An asymptotic confidence interval of $f_s(x_0)$ of level 0.95 can be taken to be

19 $$\begin{aligned} \Bigg [ \widehat{f}_{n, s}(x_0) - 2.437 n^{-2/5} \frac{1}{\widehat{\alpha }_n} \left( \frac{\widehat{f}_n(x_0)^2 \widehat{d}_n(x_0) }{24}\right) ^{1/5}, \nonumber \\ \widehat{f}_{n, s}(x_0) + 2.337 n^{-2/5} \frac{1}{\widehat{\alpha }_n} \left( \frac{\widehat{f}_n(x_0)^2 \widehat{d}_n(x_0) }{24}\right) ^{1/5} \Bigg ]. \end{aligned}$$

##### Case 3

In this case, we assume that the curve $f_0$ admits a linear portion. For $x_0 \in (0,1)$, let [*a*, *b*] be the closure of the largest interval containing $x_0$ on which $f_0$ is linear. Then, from Theorem 2.11 of Chen and Wellner (2016), it follows that

$$\begin{aligned} \left( \begin{array}{ll} n^{1/2} \left( \widehat{f}_{n,s}(x_0) - f_s(x_0) \right) \\ n^{1/2} \left( \widehat{f}'_{n,s}(x_0) - f'_s(x_0) \right) \end{array} \right) \rightarrow _d \frac{1}{\alpha _0} \left( \begin{array}{ll} \mathbb {H}''_*(x_0) \\ \mathbb {H}^{(3)}_*(x_0) \end{array} \right) \end{aligned}$$

where $\mathbb {H}_*$ is a stochastic process which is different from $\mathbb {H}$ introduced above in case 1. For more details about $\mathbb {H}_*$ we refer to Chen and Wellner (2016). To construct confidence intervals for $f_s(x_0)$ we resort again to the *m* out of *n* bootstrap. The procedure is very analogous to the one described for the monotone constraint. To sample from the convex LSE, we recall that this estimator is a piecewise linear convex density with kink points occurring between the observations (see Corollary 2.1 of Groeneboom et al., 2001). Thus,

$$\begin{aligned} \widehat{f}_n(x) = c_1 \frac{2 (\tau _1 - x)_+}{\tau ^2_1} + \ldots + c_p \frac{2 (\tau _p - x)_+}{\tau ^p_1} \end{aligned}$$

where $z_+ = \max (z, 0)$, $0< \tau _1<...< \tau _p < 1$ and $c_i > 0, i=1, \ldots , p$ such that $\sum _{i=1}^p c_i =1$. Thus, $\widehat{f}_n$ can be viewed as a mixture of *m* rescaled Beta’s with parameters 1 and 2 from which we sample *B* new data as done in the monotone case above. For each of the *B* bootstrapped samples, we compute the convex LSE and the estimator of the density of the signal, $\hat{f}^*_{n, s}$. We again conjecture that the distribution of $m^{1/2}_n ( \hat{f}^*_{n, s}(x_0) - \hat{f}_{n, s}(x_0))$ and that of $n^{1/2} (\hat{f}_{n, s}(x_0) - f_s(x_0)$ converge to the same limit as $n \rightarrow \infty$ provided that $m_n = o(n)$.

**Table 10:** Empirical coverage of the asymptotic confidence interval of $f_s(x_0)$ for the Setting #3 based on $m_n$ out of *n* bootstrapping from the Grenander estimator with $m_n = \lfloor n^{4/5} \rfloor$

$$x_0 $$	Setting #3		
	$n=1000$	$n=5000$	$n=10000$
0.4	96	94	91
0.5	93	89	88

The number of replications was taken to be $M=100$ while the quantiles of the bootstrapped distribution were estimated using $B=500$ bootstrap samples

**Table 11:** Empirical coverage of the asymptotic confidence interval of $f_s(x_0)$ as given in Eq. 19 for the Setting #4

$$x_0 $$	Setting #4		
	$n=1000$	$n=5000$	$n=10000$
0.2	98	96	95
0.3	99	97	96

In estimating the second derivative $f''_0(x_0)$ we took $c= 0.5$. Also, the number of replications was chosen to be $M=100$

Figures 9 and 10 depict 5 independent convex LSE’s of the signal density for the Settings #3 and #4. In Setting #3, the signal density is linear on its support, and this is reason why the estimators have very few changes of slope. In Setting #4, we see more kink points as the signal density is strictly convex. Note that in both cases, the convergence of the estimator we propose is very good for $n =10000$.

In Tables 10 and 11 we report the values of the empirical coverages of the confidence intervals described above for some chosen points $x_0$. In the former setting, we use the *m* out of *n* bootstrap with $m= m_n = \lfloor n^{4/5} \rfloor$. To estimate the second derivative of the true mixed density, we took $c=0.5$. We find the results to be overall quite satisfactory.

#### Estimation of the Signal Density under Log-concavity

In this section, we consider the situation where $F_s$ admits a log-concave density, $f_s$, with respect to Lebesgue measure. Maximum likelihood estimation under the log-concave constraint has been considered by many authors; see among others, Balabdaoui et al. (2009a); Rufibach (2007); Doss and Wellner (2016); Dümbgen and Rufibach (2009) and Balabdaoui et al. (2009b). In a nutshell, $f_s$ is said to be log-concave if $\log (f_s)$ is a proper concave function. In Dümbgen and Rufibach (2009), it was shown that the MLE exists, is unique and its logarithm is piecewise linear with break points at some of the observations. An efficient active set algorithm was developed by Dümbgen et al. (2007) to compute the estimator. In Balabdaoui and Besdziek (2024), the convergence properties of the (pseudo) log-concave MLE of the unknown component in a semi-parametric mixture of a standard Gaussian background and a log-concave signal were studied. In that work, $\alpha _0$ was estimated using the method of Patra and Sen (2016). In this section, we put ourselves in a similar setting, that is

$$\begin{aligned} f_0(x) = (1-\alpha _0) \varphi (x) + \alpha _0 f_s(x) \end{aligned}$$

where $\varphi$ is the density of a standard Gaussian and $f_s$ is log-concave with support strictly included in $\mathbb {R}$. We further assume that we are in the situation of Problem #1; i.e., that Assumption A2 is satisfied. Let $\widehat{\alpha }_n$ and $\widehat{a}_n$ be our estimators of $\alpha _0$ and $a_0$. We denote by $\widehat{f}_{n, s}$ the log-concave MLE of $f_s$ defined as

$$\begin{aligned} \text {argmax}_{\psi \in \mathcal {C}_1} \frac{1}{n} \sum _{j=1}^n \log \left( (1-\widehat{\alpha }_n) \varphi (X_j) + \widehat{\alpha }_n e^{\psi (X_j)} \right) \end{aligned}$$

where $\mathcal {C}_1$ is the set of concave functions such that $e^{\psi }$ is a density for $\psi \in \mathcal {C}_1$. Since $\widehat{\alpha }_n$ converges to the truth at the $\sqrt{n}$-rate, Theorem 4.5 of Balabdaoui and Besdziek (2024) applies, and we have

$$\begin{aligned} \int \left| \widehat{f}_{n, s}(x) - f_s(x) \right| dx = O_{\mathbb {P}}(n^{-2/5}). \end{aligned}$$

Furthermore, we conjecture here as well that if $f_s$ is twice-continuously differentiable in a neighborhood of some point $x_0$ such that $f''_s(x_0) \ne 0$, it holds that

$$\begin{aligned} n^{2/5} (\widehat{f}_{n, s}(x_0) - f_0(x_0)) \rightarrow _d \mathbb {D}(x_0) \end{aligned}$$

where $\mathbb {D}(x_0)$ is a well-defined real random variable. One can conjecture that $\mathbb {D}(x_0) = c(x_0) \mathbb {H}''(0)$, where $\mathbb {H}''(0)$ is the same as above. However, the deterministic constant $c(x_0)$ seems to be difficult to pin down. In case $f''_s(x_0) =0$, the asymptotic theory of the estimator is yet to be established. In this section, we will only present simulation results based on different samples from the mixed density. We believe that it is possible to use either a bootstrap technique or estimate the constant $c(x_0)$ under the conjectured asymptotic distribution in the case $f''(x_0) \ne 0$. However, in the lack of any knowledge about the deterministic constant $c(x_0)$, it is unclear how such a confidence interval should be built in this case.

As in Balabdaoui and Besdziek (2024), we use the EM algorithm to compute the log-concave (pseudo) MLE $\widehat{f}_{n,s}$. The only difference is that we use our estimator $\widehat{\alpha }_n$ instead of the one constructed by Patra and Sen (2016). In the M-step, we use the R-package **logcondens** which can compute a log-concave MLE, given weights. We refer to Rufibach (2007). To give more details, consider $\Delta = (\Delta _1,...,\Delta _n)$ to be the vector of 0/1 indicating whether a data point comes from the background or from the signal; i.e., $\Delta _i = 1$ if the *i*th observation comes from the background distribution and $\Delta _i = 0$ if it comes from the signal distribution. The (pseudo) likelihood function at a log-concave density *g* based on the *complete data* $(X_1, \ldots , X_n, \Delta _1, \ldots , \Delta _n)$ is given by

$$\begin{aligned} \ell _n(g | X_1,...,X_n, \Delta _1, \ldots , \Delta _n)= &  n^{-1} \sum _{i=1}^n \Big [\Delta _i \log (\varphi (X_i)) +(1-\Delta _i)\log (g(X_i)) \\ &  \ \ \ \ + \Delta _i \log (1-\alpha _n) +(1-\Delta _i)\log (\alpha _n) \Big ]. \end{aligned}$$

The EM algorithm is initialized by finding the MLE of a Gaussian distribution with parameters $\mu$ and $\sigma ^2$. For this step, we used the R-function normalmixEM. Let $\widehat{g}^{(0)}_n$ be the corresponding Gaussian density, and more generally $\widehat{g}_n^{(k)}$ the obtained log-concave maximizer at the *k*-th iteration. Then, the algorithm alternates between the E-step where we compute the weights

$$\begin{aligned} \gamma _i^{(k+1)} = \frac{(1-\alpha _n)\varphi (X_i)}{(1-\hat{\alpha }_n)\varphi (X_i) + \alpha _n \widehat{g}_n^{(k)}(X_i)}, \ i=1, \ldots , n \end{aligned}$$

for $k \in \{0, \ldots , k_{max} \}$, with $k_{max}$ the maximal number of iterations we allow for the (pseudo) EM algorithm, and the M-step where we compute the log-concave (pseudo) MLE $\widehat{g}^{(k)}_n = e^{\widehat{\psi }^{(k)}_n}$, where $\widehat{\psi }^{(k)}_n$ maximizes the functional

$$\begin{aligned} \psi \mapsto \ \sum _{i=1}^n w^{(k)}_i \psi (X_i) - \int _{\mathbb {R}} e^{\psi (x)} dx \end{aligned}$$

over concave functions $\psi$, for $k \in \{1, \ldots , k_{max} \}$ and

$$\begin{aligned} w^{(k)}_i = \frac{1-\gamma ^{(k)}_i}{n - \sum _{i=1}^n \gamma ^{(k)}_i}, \ i=1, \ldots , n. \end{aligned}$$

With $k_{max} =250$ and tol= 5E-06, we require as our stopping rule (in case $k+1 < k_{max}$) that $\max _{i=1,...,n} \big \vert \gamma _i^{(k+1)}-\gamma _i^{(k)} \big \vert <\texttt {tol}$.

**Fig. 11:**
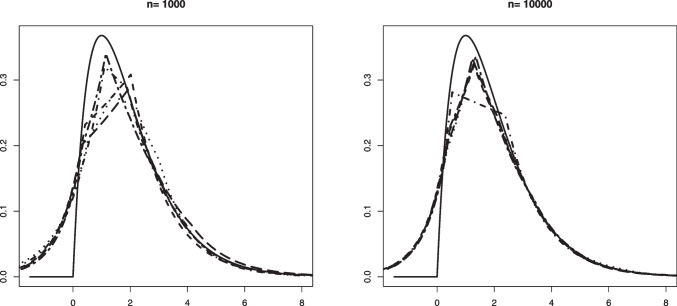
An example in Setting #5 of the (pseudo) log-concave MLE of the density of Gamma(2,1) (depicted by the solid line). The figure shows 5 such estimators based on independent samples of size $n=1000$ (left) and $n=10000$ (right). The mixing proportion is $\alpha _0 = 0.8$

**Fig. 12:**
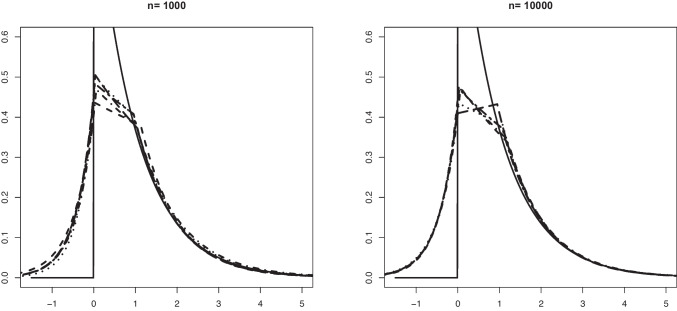
An example in Setting #6 of the (pseudo) log-concave MLE of the density of Exp(1) (depicted by the solid line). The figure shows 5 such estimators based on independent samples of size $n=1000$ (left) and $n=10000$ (right). The mixing proportion is $\alpha _0 = 0.8$

**Fig. 13:**
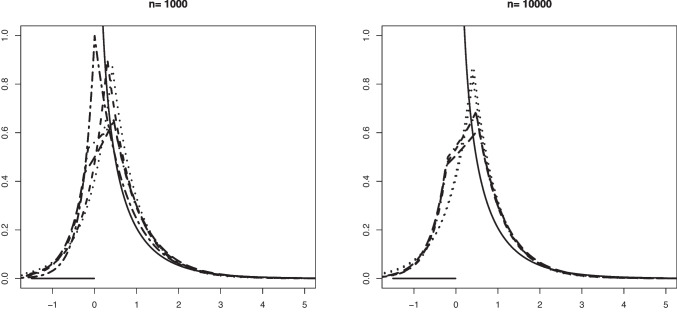
An example in Setting #7 of the (pseudo) log-concave MLE of the density of Gamma(1/2,1) (depicted by the solid line). The figure shows 5 such estimators based on independent samples of size $n=1000$ (left) and $n=10000$ (right). The mixing proportion is $\alpha _0 = 0.8$

Figures 11, 12 and 13 show 5 independent (pseudo) log-concave MLE’s of the signal density for the Settings #5, #6 and #7. In Settings #6 and #7, the signal density has its maximum at $a_0$; in Setting #7, this maximum is infinity. In all three settings, the estimator matches the true density very well for all values that are to the right of $a_0$ and have some distance to it. This is already the case for $n=1000$. However, for values that are to the left of $a_0$ and in particular for the maximal value in Settings #6 and #7, the fit is less good. However, the fit can be much improved for larger sample sizes. This is illustrated by the MLE plots of Figure B1 of the supplementary material obtained for Setting #6 and $n =10^5$.

### Application to Prostate Cancer Data

The prostate cancer data consist of a $6033 \times 102$ matrix. Each row $i = 1, \ldots , 6033$ gives the expression level of gene *i* for $j=1, \ldots , 102$ men: Healthy control subjects for $j=1, \ldots , 50$ and cancer patients for $j = 51, \ldots , 102$. Let $x_{ij}$ denote the expression level for gene $i =1, \ldots , 6033$ and man $j =1, \ldots , 50$ or $j = 51, \ldots , 102$ depending on whether the patient is healthy or not. Following the methodology used in Patra and Sen (2016) we compute the two-sided p-values based on the absolute values of the (asymptotic) t-statistics

$$\begin{aligned} t_i = \frac{\bar{x}_i(1) - \bar{x}_i(2)}{s_i} \end{aligned}$$

where

$$\begin{aligned} \bar{x}_i(1) = \frac{1}{50} \sum _{j=1}^{50} x_{ij}, \ \ \bar{x}_i(2) = \frac{1}{52} \sum _{j=51}^{102} x_{ij}, \end{aligned}$$

and $s_i$ is the standard error estimate whose squared value is given by

$$\begin{aligned} s^2_i = \left( \frac{1}{50} + \frac{1}{52}\right) \frac{\sum _{j=1}^{50} \left( x_{ij} -\bar{x}_i(1)\right) ^2 + \sum _{j=51}^{102} \left( x_{ij} -\bar{x}_i(2)\right) ^2 }{100} \end{aligned}$$

for $i=1, \ldots , 6033$. Under the assumption that $t_i, i=1, \ldots , 6033$ are i.i.d. (note this assumption was also made and defended in Patra and Sen, 2016), these values have asymptotically a t-distribution with 100 degrees of freedom. If $F_{t_{100}}$ denotes the latter distribution, then the p-values $p_i, i =1, \ldots , 6033$ are given by

$$\begin{aligned} p_i = 2 (1 - F_{t_{100}}(\vert t_i \vert )). \end{aligned}$$

**Fig. 14:**
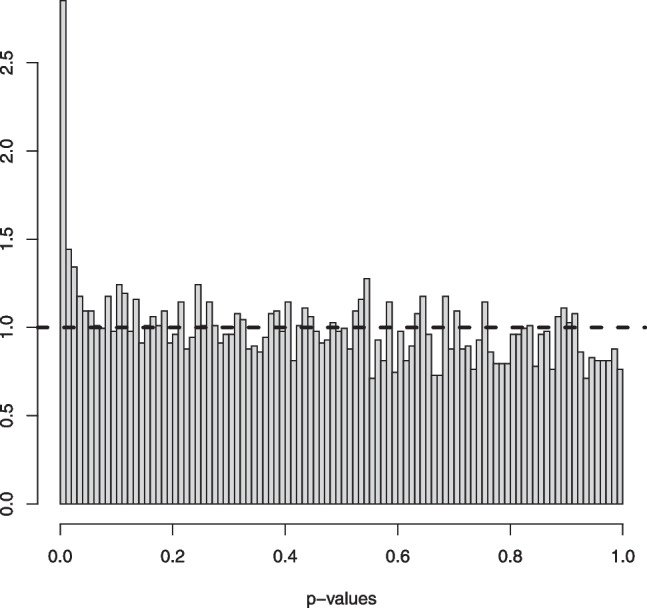
Histogram of the p-values computed for the prostate cancer data. See text for details. The dashed line corresponds to the uniform distribution on [0, 1]

The histogram of these p-values is shown in Fig. 14, which has a very similar shape to the one shown in (Patra and Sen 2016, Figure 5-a). To make a link to the scope of this paper, we assume that these p-values come from the mixture model

$$\begin{aligned} f_0(x) = (1-\alpha _0) + \alpha _0 f_s(x), x \in [0,1] \end{aligned}$$

where $\alpha _0 \in (0,1)$ and $f_s$ is a non-increasing density with support $[0, a_0]$ for some unknown $a_0 \in (0,1)$.

Since we do not have a clear idea about what to take for $\kappa _0$, we can compute $\widehat{\kappa }_n$ as described In Section 3.4. Recall that the signal density in this problem is the density of the distribution of the p-values under the alternative. For this reason, it seems reasonable to assume that $a_0$ is small. Here, we search for $\widehat{\kappa }_n$ in the grid $\{0.01, 0.02, \ldots , 0.30\}$, and find $\widehat{\kappa }_n =0.11$. This yields $\widehat{\alpha }_n = 0.0382$.

In order to construct confidence intervals for the true mixing proportion $\alpha _0$, we resort to a resampling technique in which we randomly select *B* times from each of the two groups 45 out of the 50 and 52 men respectively. For $b =1, \ldots , B$, the obtained $t^{(b)}_i$ is computed in a similar way as above except that the estimate of the standard deviation is now given by

$$\begin{aligned} s^{(b)}_i = \frac{2}{45} \frac{\sum _{j \in I^{(b)}_1} \left( x_{ij} -\bar{x}^{(b)}_i(1)\right) ^2 + \sum _{j \in I^{(b)}_2} \left( x_{ij} -\bar{x}^{(b)}_i(2)\right) ^2 }{88} \end{aligned}$$

where $I^{(b)}_1$ and $I^{(b)}_2$ are the indices corresponding to the selected men in the healthy and unhealthy group respectively, and $\bar{x}^{(b)}_i(1)$ and $\bar{x}^{(b)}_i(2)$ are the mean values of the expression levels in each of the selected sub-groups. Note also that in this case the p-value is

$$\begin{aligned} p^{(b)}_i = 2 (1 - F_{t_{88}}(\vert t^{(b)}_i \vert )). \end{aligned}$$

**Fig. 15:**
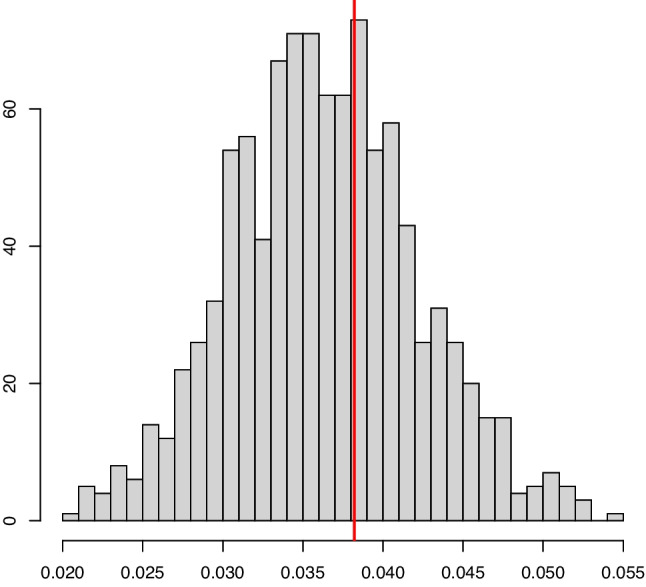
Histogram of $\widehat{\alpha }^{(b)}_n, b=1, \ldots , 1000$. See text for details. The vertical red line depicts $\widehat{\alpha }_n = 0.0382$ computed using the whole dataset

**Table 12:** Statistics for the obtained $\widehat{\alpha }^{(b)}_n, b =1, \ldots , 1000$ using resampling

Mean	Median	Standard deviation	95%-CI
0.0363	0.0362	0.0058	[0.0253, 0.0479]

for $i=1, \ldots , 6033$. For $b =1, \ldots , B$ we compute the estimator $\widehat{\alpha }^{(b)}_n$ using the obtained dataset of p-values. Here, we use the same value $\widehat{\kappa }_n = 0.11$ in all the replications. For $B = 1000$, we obtain the histogram shown in Fig. 15. In Table 12 we report the mean, median, standard deviation as well as the symmetric 95%-confidence interval. In (Patra and Sen 2016, Table 6), the authors report the values of the estimators considered in their paper. Expect for Efron’s estimator (see also Efron, 2007), all the estimators fall in our 95%-confidence interval.

**Fig. 16:**
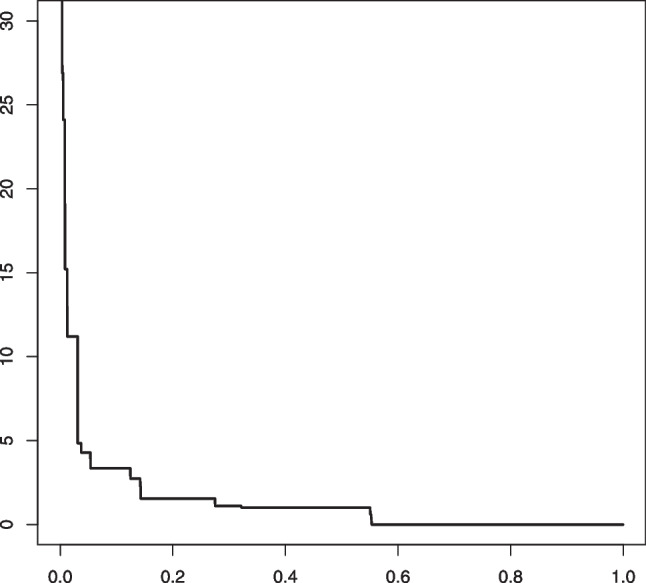
The monotone estimator $\widehat{f}_{n,s}$ of the unknown signal density of the p-values for the prostate cancer data

Using the assumed monotonicity of $f_s$, we can compute the density estimator $\widehat{f}_{n,s}$ introduced in Eq. 13. This estimator is shown is Fig. 16. The value of $\widehat{f}_{n,s}$ at the smallest p-value ($\approx 1.54 10^{-7}$) is very large ($\approx 2800$) and hence the plot of $\widehat{f}_{n,s}$ was truncated at the value 30. Note that our estimator shares some similarities with the one shown in (Patra and Sen 2016, Figure 5 - d).

## Conclusions and Discussion

In this work, we introduced a new and simple estimator $\hat{\alpha }_n$ for the mixing proportion in a two-component mixture with a known background component in the setting where the signal distribution has its support strictly to the right/left of some (unknown) value $a_0$. We proved that $\hat{\alpha }_n$ converges at the parametric rate to the true mixing proportion, and also provided its limit distribution. Based on this estimator of the mixing proportion, we constructed an estimator of the starting (resp. ending) point of the signal distribution, that is $a_0$, whose rate of convergence was proved to be depend on the behavior of the signal density at this point. Our main theory builds upon the requirement that it is known a priori that the starting (resp. ending) point of the signal distribution is to the right (resp. left) of some value $\kappa _0$. Ideally, $\kappa _0$ should be made available by an expert in the field under study, or be imposed by the problem itself. We presented a formal method to test whether a certain value $\kappa _0$ is appropriate or not. In some applications, it might be not that clear which value $\kappa _0$ to take. For this reason, we also introduced a method for the estimation of $a_0$ that does not require this pre-determined value $\kappa _0$ but instead yields an estimator $\widehat{\kappa }_n$ for the “optimal” value $\kappa _0$, which is $a_0$. Our simulations reveal that this method is very promising for some of the examples under study. Building on already established results, we also showed how our estimator $\hat{\alpha }_n$ can be applied to obtain an estimator for the density of the signal distribution under several shape constraints. Our work was supplemented by simulations that confirm our theoretical results, and applied to a real data to illustrate the usefulness of our methodology.

One referee asked us whether our work can be extended to the situation where the signal distribution is supported on $$(s_1, s_2) \subset (b_1, b_2)$$, where $$[b_1, b_2]$$ is the support of the background distribution, such that $$ b_1 < s_1$$ and $$s_2 < b_2$$. Note that this setting is related to the one added in the introduction as the forth item. Since $$b_1$$ and $$b_2$$ should be known (as the background distribution is known), the main question is whether one of the end-points $$s_1$$ or $$s_2$$ is known. In case one of these end-points is known, the problem is actually easier than the one treated in this work. In fact, let us assume that $$s_2$$ is known. Then, for all $$x \in [s_2,b_2]$$,

$$ F_0(x) = (1-\alpha _0) F_{b}(x) + \alpha _0 $$

since $F_s(x) =1$ for all *x* in this region. It follows that

20 $$\begin{aligned} \alpha _0 = 1 - \frac{1-F_0(x)}{1- F_{b}(x)} = \frac{F_0(x) - F_{b}(x) }{1- F_{b}(x)} \end{aligned}$$

for all $x \in [s_2,b_2]$. To construct an estimator for $\alpha _0$, one can take a single point, for example $x = s_2$, and replace $F_0$ by the empirical distribution function $\mathbb {F}_n$. This yields the estimator

$$ \widehat{\alpha }_n = \frac{\mathbb {F}_n(s_2) - F_{b}(s_2) }{1- F_{b}(s_2)}. $$

Note that the equation in Eq. 20 can be also integrated over $x \in [s_2, b_2]$. While this yields other types of estimators of $\alpha _0$, it is unclear whether they are more efficient than the one defined above. If both $s_1$ and $s_2$ are unknown, then it seems that one can either use the scope of Problem #1 or Problem #2, or both. In fact, if we focus on the left, and if $\kappa _0 \in (b_1, s_1)$, then for $x \ge \kappa _0$ we have that

$$\begin{aligned} 1 - \frac{F_0(x) }{F_{b}(x)} = \alpha _0 - \alpha _0 \frac{F_s(x)}{F_{b}(x)} \end{aligned}$$

which again implies that

$$ \alpha _0 = \sup _{x \ge \kappa _0} \left( 1 - \frac{F_0(x)}{F_{b}(x)} \right) = 1 - \inf _{x \ge \kappa _0} \frac{F_0(x)}{F_{b}(x)} $$

and which can be estimated as done in the paper. In the scope of Problem #2, we should consider $\kappa _0 \in (s_2, b_2)$. It would be possible to somehow merge both problems but this means that one should estimate the endpoints $s_1$ and $s_2$ or have access to two values of $\kappa _0$, $\kappa ^L_0 \in (b_1, s_1)$ and $\kappa ^R_0 \in (s_2, b_2)$ say. These values will then yield two estimators:

$$ \widehat{\alpha }^L_n = 1 - \inf _{x \ge \kappa ^L_0} \frac{\mathbb {F}_n(x)}{F_{b}(x)} $$

and

$$ \widehat{\alpha }^R_n = 1 - \inf _{x \le \kappa ^R_0} \frac{1-\mathbb {F}_n(x)}{1-F_{b}(x)}. $$

The question is now these estimators can be put together in some reasonable way. One can of course take simply the average of both or take a weighted average with the weight that minimizes some cross-validation criterion. Another setting to which the referee later pointed out is one which can be related to the case where $\mathcal {S}_b = \mathbb {R}$ and $\mathcal {S}^c_s \subset (a_1, a_2)$ for $\infty< a_1< a_2 < \infty$, where $(a_1, a_2)$ is the largest interval with such a property (see setting #3 on page 3). This situation is different because the set on which the signal is known not to be supported is located “in the middle”, and in this case,

$$ \inf _{F_b(x) > 0} \frac{F_s(x)}{F_b(x)} = \inf _{ x \in \mathbb {R}} \frac{F_s(x)}{F_b(x)} $$

might not be equal 0. Knowledge of either $a_1$ or $a_2$ can help in circumventing the problem. Since the distribution function $F_s$ is constant on $[a_1, a_2]$, it holds that

$$ F_0(x) - F_0(a_1) = (1-\alpha _0) (F_b(x) - F_b(a_1)) $$

and hence

$$ \alpha _0 = 1- \frac{F_0(x) - F_0(a_1)}{F_b(x) - F_b(a_1)} $$

for all $x \in [a_1, a_2]$. Although this is a promising identity, one needs to have a lower bound on $a_2 - a_1$ to be able to use an *x* which indeed belongs to $[a_1, a_2]$. If $a_2- a_1 > \eta$ for some known $\eta > 0$, then we can consider constructing an estimator of $\alpha _0$ which uses the process

$$\begin{aligned} \left\{ 1- \frac{\mathbb {F}_n(x) - \mathbb {F}_n(a_1)}{F_b(x) - F_b(a_1)}, x \in [a_1, a_1 + \eta ] \right\} \end{aligned}$$

in some optimal way. The situation becomes much more complicated when both $a_1$ and $a_2$ are unknown as the problem significantly deviates from the scope of this work.

## Supplementary Information

Below is the link to the electronic supplementary material.

Supplementary file 1 (pdf 880 KB)
