# Supplementary material for: Simple Estimators of the Mixing Proportion in a Semi-Parametric Mixture with Known Component
Source: Sankhya Ser A. 2025 Oct 22;88(1):80–135. doi: 10.1007/s13171-025-00421-w (PMC12967557; doi:10.1007/s13171-025-00421-w)
Supplement: Supplementary file 1 — (pdf 880 KB) [file 13171_2025_421_MOESM1_ESM.pdf]

Supplementary material

Fadoua Balabdaoui[1*] and Harald Besdziek[1]

[1*]Seminar für Statistik, ETH Zürich, Rämistrasse 101, 8092 Zürich, Switzerland.

*Corresponding author(s). E-mail(s): fadoua.balabdaoui@stat.math.ethz.ch;

Abstract

In this supplement we provide additional proofs.

Keywords: asymptotic theory, Brownian process, mixture distribution, semi-parametric

1 Proof of Theorem 2.1

We can write

$$\begin{aligned}\frac{\mathbb{F}_n(x)}{F_b(x)} &= \frac{\mathbb{F}_n(x) - F_0(x)}{F_b(x)} + \frac{F_0(x)}{F_b(x)} \\ &= \frac{1}{\sqrt{n}} \frac{\sqrt{n}\left(\mathbb{F}_n(x) - F_0(x)\right)}{F_b(x)} + 1 - \alpha_0 + \alpha_0 \frac{F_s(x)}{F_b(x)}\end{aligned}$$

and hence

$$\widehat{\alpha}_n = 1 - \inf_{x \ge \kappa_0} \frac{\mathbb{F}_n(x)}{F_b(x)} = \alpha_0 - \inf_{x \ge \kappa_0} \left\{ \frac{1}{\sqrt{n}} \frac{\sqrt{n}\left(\mathbb{F}_n(x) - F_0(x)\right)}{F_b(x)} + \alpha_0 \frac{F_s(x)}{F_b(x)} \right\}$$

which is equivalent to writing that

$$\sqrt{n}(\widehat{\alpha}_n - \alpha_0) = -\inf_{x \ge \kappa_0} \left\{ \frac{\sqrt{n}\left(\mathbb{F}_n(x) - F_0(x)\right)}{F_b(x)} + \alpha_0 \frac{\sqrt{n} F_s(x)}{F_b(x)} \right\}$$

$$= -\min\left\{ \inf_{x\in[\kappa_0,a_0)} \left\{ \frac{\sqrt{n}\,(\mathbb{F}_n(x)-F_0(x))}{F_b(x)} \right\} \right.$$
$$\left. , \inf_{x\in[a_0,\infty)} \left\{ \frac{\sqrt{n}\,(\mathbb{F}_n(x)-F_0(x))}{F_b(x)} + \alpha_0 \frac{\sqrt{n}F_s(x)}{F_b(x)} \right\} \right\}.$$

Using the Hungarian Embedding (see Komlos, Major, and Tusnády (1975)), we can find a probability space, a standard Brownian Bridge $\mathbb{B}^*$ and i.i.d. random variables $X_1^*, \ldots, X_n^* \sim F_0$ defined on this probability space such that

$$\left\| \sqrt{n}(\mathbb{F}_n^* - F_0) - \mathbb{B}^* \circ F_0 \right\|_\infty \le C \frac{\log n}{\sqrt{n}}, \quad \text{almost surely} \tag{1}$$

where C is some fixed positive constant, and $\mathbb{F}_n^*$ is the empirical cdf based on $X_1^*, \ldots, X_n^*$. Since the probability statements remain unchanged as to whether we work with $\mathbb{F}_n$ or $\mathbb{F}_n^*$, we will simply assume that $\mathbb{F}_n$ satisfies the preceding property and will also write $\mathbb{B}$ for $\mathbb{B}^*$. We have that

$$\begin{aligned}\sup_{x\ge\kappa_0} \left| \frac{\sqrt{n}(\mathbb{F}_n(x)-F_0(x))}{F_b(x)} - \frac{\mathbb{B}\circ F_0(x)}{F_b(x)} \right| &\le \frac{1}{F_b(\kappa_0)} \left\| \sqrt{n}(\mathbb{F}_n - F_0) - \mathbb{B}\circ F_0 \right\|_\infty \\ &\le \frac{D\log n}{\sqrt{n}}, \quad \text{almost surely}\end{aligned} \tag{2}$$

using the result given in (1) (after the notational adjustments), and where $D = C/F_b(\kappa_0)$. Fix $\epsilon > 0$ and let $\lambda = \lambda_\epsilon > 0$ such that

$$\begin{aligned}P\left(\sup_{x\ge\kappa_0} \frac{|\mathbb{B}\circ F_0(x)|}{F_b(x)} > \lambda \right) &\le P\left(\sup_{x\ge\kappa_0} |\mathbb{B}\circ F_0(x)| > \lambda F_b(\kappa_0) \right) \\ &\le P\left(\|\mathbb{B}\|_\infty > \lambda F_b(\kappa_0) \right), \quad \text{using the fact that } \|\mathbb{B}\|_\infty = \|\mathbb{B}\circ F_0\|_\infty \\ &= 2\sum_{k=1}^{\infty} (-1)^{k+1} \exp(-2k^2\lambda^2 F_b^2(\kappa_0)) \le \epsilon\end{aligned}$$

where the last equality can be shown via reflection methods; see e.g., Shorack and Wellner (2009). Let $\mathcal{N}$ be the null set on which the inequality in (2) is not fulfilled. Define now the event

$$\mathcal{E} = \mathcal{N}^c \cap \left\{ \omega : \sup_{x\ge\kappa_0} \frac{|\mathbb{B}\circ F_0(x)|}{F_b(x)} \le \lambda \right\}.$$

Using (2) it follows that for n large enough such that $D\log n/\sqrt{n} \le \lambda$

$$\mathcal{E} \subset \left\{ \omega : \sup_{x\ge\kappa_0} \left| \frac{\sqrt{n}(\mathbb{F}_n(x)-F_0(x))}{F_b(x)} \right| \le 2\lambda \right\}.$$

Let $C_n \subset [a_0, \infty)$ such that for all $x \in C_n$

$$F_s(x) > \frac{4\lambda}{\alpha_0 \sqrt{n}}.$$

Then, for all $x \in C_n$ and $\omega \in \mathcal{E}$, we have that

$$\frac{\sqrt{n}\,(\mathbb{F}_n(x) - F_0(x))}{F_b(x)} + \alpha_0 \frac{\sqrt{n} F_s(x)}{F_b(x)} > -2\lambda + 4\lambda = 2\lambda.$$

On the other hand, for $\omega \in \mathcal{E}$ and $x \in [\kappa_0, a_0)$ we have that

$$\frac{\sqrt{n}\,(\mathbb{F}_n(x) - F_0(x))}{F_b(x)} \le \frac{\sqrt{n}\,|\mathbb{F}_n(x) - F_0(x)|}{F_b(x)} \le 2\lambda.$$

It follows that for $\omega \in \mathcal{E}$

$$\begin{aligned}
&\min\left\{ \inf_{x \in [\kappa_0, a_0)} \left\{ \frac{\sqrt{n}\,(\mathbb{F}_n(x) - F_0(x))}{F_b(x)} \right\}, \inf_{x \in [a_0, \infty)} \left\{ \frac{\sqrt{n}\,(\mathbb{F}_n(x) - F_0(x))}{F_b(x)} + \alpha_0 \frac{\sqrt{n} F_s(x)}{F_b(x)} \right\} \right\} \\
&= \min\left\{ \inf_{x \in [\kappa_0, a_0)} \left\{ \frac{\sqrt{n}\,(\mathbb{F}_n(x) - F_0(x))}{F_b(x)} \right\}, \inf_{x \in [a_0, a_0 + h_n]} \left\{ \frac{\sqrt{n}\,(\mathbb{F}_n(x) - F_0(x))}{F_b(x)} + \alpha_0 \frac{\sqrt{n} F_s(x)}{F_b(x)} \right\} \right\}
\end{aligned}$$

where $(h_n)_{n \ge 1}$ is a non-negative sequence converging to 0 such that

$$[a_0, a_0 + h_n] = C_n^c = \left\{ x \ge a_0 : F_s(x) \le \frac{4\lambda}{\alpha_0 \sqrt{n}} \right\}, \quad \text{or equivalently} \quad F_s(a_0 + h_n) = \frac{4\lambda}{\alpha_0 \sqrt{n}}.$$

Now, fix $c \in [0, 4\lambda/\alpha_0]$. For $\omega \in \mathcal{E}$ and $x_{n,c} \in [a_0, a_0 + h_n]$ such that $F_s(x_{n,c}) = c/\sqrt{n}$ we have that

$$\begin{aligned}
&\frac{\sqrt{n}\,(\mathbb{F}_n(x_{n,c}) - F_0(x_{n,c}))}{F_b(x_{n,c})} + \alpha_0 \frac{\sqrt{n} F_s(x_{n,c})}{F_b(x_{n,c})} \\
&\quad = \frac{\sqrt{n}\,(\mathbb{F}_n(x_{n,c}) - F_0(x_{n,c}))}{F_b(x_{n,c})} + \alpha_0 \frac{c}{F_b(x_{n,c})} \\
&\quad = \frac{\sqrt{n}\,(\mathbb{F}_n(x_{n,c}) - F_0(x_{n,c}))}{F_b(x_{n,c})} - \frac{\mathbb{B} \circ F_0(x_{n,c})}{F_b(x_{n,c})} + \frac{\mathbb{B} \circ F_0(x_{n,c})}{F_b(x_{n,c})} \\
&\qquad + \alpha_0 \frac{c}{F_b(x_{n,c})} \\
&\quad = \frac{\sqrt{n}\,(\mathbb{F}_n(x_{n,c}) - F_0(x_{n,c}))}{F_b(x_{n,c})} - \frac{\mathbb{B} \circ F_0(x_{n,c})}{F_b(x_{n,c})} + \frac{\mathbb{B} \circ F_0(x_{n,c})}{F_b(x_{n,c})} - \frac{\mathbb{B} \circ F_0(a_0)}{F_b(a_0)} \\
&\qquad + \frac{\mathbb{B} \circ F_0(a_0)}{F_b(a_0)} + \frac{\alpha_0 c}{F_b(a_0)} + \alpha_0 c \left(\frac{1}{F_b(x_{n,c})} - \frac{1}{F_b(a_0)} \right).
\end{aligned}$$

Using (2) and $\sqrt{n}F_s(x_{n,c}) = c$, it follows that

$$\left|\frac{\sqrt{n}\,(\mathbb{F}_n(x_{n,c}) - F_0(x_{n,c}))}{F_b(x_{n,c})} + \alpha_0\frac{\sqrt{n}F_s(x_{n,c})}{F_b(x_{n,c})} - \frac{\mathbb{B}\circ F_0(a_0)}{F_b(a_0)} - \frac{\alpha_0 c}{F_b(a_0)}\right|$$
$$\leq \frac{D\log n}{\sqrt{n}} + \left|\frac{\mathbb{B}\circ F_0(x_{n,c})}{F_b(x_{n,c})} - \frac{\mathbb{B}\circ F_0(a_0)}{F_b(a_0)}\right| + \alpha_0 c\left|\frac{1}{F_b(x_{n,c})} - \frac{1}{F_b(a_0)}\right|.$$

Using continuity of $\mathbb{B}$ and F_b and the fact that c belongs to a compact, it holds for all $\omega \in \mathcal{E}$ that

$$\sup_{c\in[0,4\lambda/\alpha_0]}\left\{\left|\frac{\mathbb{B}\circ F_0(x_{n,c})}{F_b(x_{n,c})} - \frac{\mathbb{B}\circ F_0(a_0)}{F_b(a_0)}\right| + \alpha_0 c\left|\frac{1}{F_b(x_{n,c})} - \frac{1}{F_b(a_0)}\right|\right\} \to 0$$

almost surely as $n \to \infty$, implying that

$$\frac{\sqrt{n}\,(\mathbb{F}_n(x_{n,c}) - F_0(x_{n,c}))}{F_b(x_{n,c})} + \alpha_0\frac{\sqrt{n}F_s(x_{n,c})}{F_b(x_{n,c})} = \frac{\mathbb{B}\circ F_0(a_0)}{F_b(a_0)} + \frac{\alpha_0 c}{F_b(a_0)} + o(1)$$

almost sutely, where $o(1)$ does not depend on c. Since

$$\inf_{x\in[a_0,a_0+h_n]}\left\{\frac{\sqrt{n}\,(\mathbb{F}_n(x) - F_0(x))}{F_b(x)} + \alpha_0\frac{\sqrt{n}F_s(x)}{F_b(x)}\right\}$$
$$= \inf_{c\in[0,4\lambda/\alpha_0]}\left\{\frac{\sqrt{n}\,(\mathbb{F}_n(x_{n,c}) - F_0(x_{n,c}))}{F_b(x_{n,c})} + \alpha_0\frac{\sqrt{n}F_s(x_{n,c})}{F_b(x_{n,c})}\right\}$$

it follows that for all $\omega \in \mathcal{E}$

$$\inf_{x\in[a_0,a_0+h_n]}\left\{\frac{\sqrt{n}\,(\mathbb{F}_n(x) - F_0(x))}{F_b(x)} + \alpha_0\frac{\sqrt{n}F_s(x)}{F_b(x)}\right\}$$
$$\to \inf_{c\in[0,4\lambda/\alpha_0]}\left\{\frac{\mathbb{B}\circ F_0(a_0)}{F_b(a_0)} + \frac{\alpha_0 c}{F_b(a_0)}\right\} = \frac{\mathbb{B}\circ F_0(a_0)}{F_b(a_0)}.$$

Using again (2), we have that

$$\inf_{x\in[\kappa_0,a_0)}\left\{\frac{\sqrt{n}\,(\mathbb{F}_n(x) - F_0(x))}{F_b(x)}\right\} \to \inf_{x\in[\kappa_0,a_0]}\frac{\mathbb{B}\circ F_0(x)}{F_b(x)} \leq \frac{\mathbb{B}\circ F_0(a_0)}{F_b(a_0)}.$$

Hence, we conclude that for $\omega \in \mathcal{E}$

$$\sqrt{n}(\widehat{\alpha}_n - \alpha_0) = -\inf_{x\geq\kappa_0}\left\{\frac{\sqrt{n}\,(\mathbb{F}_n(x) - F_0(x))}{F_b(x)} + \alpha_0\frac{\sqrt{n}F_s(x)}{F_b(x)}\right\}$$
$$\to -\inf_{x\in[\kappa_0,a_0]}\frac{\mathbb{B}\circ F_0(x)}{F_b(x)} =: \mathbb{V}. \tag{3}$$

For $t \in \mathbb{R}$, we can write that

$$P\Big(\sqrt{n}(\widehat{\alpha}_n - \alpha_0) \le t\Big) = P\Big(\sqrt{n}(\widehat{\alpha}_n - \alpha_0) \le t, \omega \notin \mathcal{E}\Big) + P\Big(\sqrt{n}(\widehat{\alpha}_n - \alpha_0) \le t, \omega \in \mathcal{E}\Big)$$
$$= I_n + II_n.$$

First, note $I_n \le P(\omega \notin \mathcal{E}) \le \epsilon$. Second, we can write that

$$II_n = P\Big(\sqrt{n}(\widehat{\alpha}_n - \alpha_0) \le t, \mathbb{V} \le t, \omega \in \mathcal{E}\Big) + P\Big(\sqrt{n}(\widehat{\alpha}_n - \alpha_0) \le t, \mathbb{V} > t, \omega \in \mathcal{E}\Big)$$
$$= P\Big(\sqrt{n}(\widehat{\alpha}_n - \alpha_0) \le t, \mathbb{V} < t, \omega \in \mathcal{E}\Big) + P\Big(\sqrt{n}(\widehat{\alpha}_n - \alpha_0) \le t, \mathbb{V} > t, \omega \in \mathcal{E}\Big)$$

using the fact that the distribution of $\mathbb{V}$ is continuous; see Lemma A.1 in the Appendix. Now, using the convergence in (3), it holds that $\{\mathbb{V} < t, \omega \in \mathcal{E}\} \subset \{\sqrt{n}(\widehat{\alpha}_n - \alpha_0) \le t, \omega \in \mathcal{E}\}$ and hence

$$P\Big(\sqrt{n}(\widehat{\alpha}_n - \alpha_0) \le t, \mathbb{V} < t, \omega \in \mathcal{E}\Big) = P\Big(\mathbb{V} < t, \omega \in \mathcal{E}\Big) = P\Big(\mathbb{V} \le t, \omega \in \mathcal{E}\Big)$$

using again Lemma A.1. On the other hand, there exists $\epsilon > 0$ such that $\{\mathbb{V} > t\} \Leftrightarrow \{\mathbb{V} \ge t + \epsilon\}$. Thus,

$$P\Big(\sqrt{n}(\widehat{\alpha}_n - \alpha_0) \le t, \mathbb{V} > t, \omega \in \mathcal{E}\Big) = P\Big(\sqrt{n}(\widehat{\alpha}_n - \alpha_0) \le t, \mathbb{V} \ge t + \epsilon, \omega \in \mathcal{E}\Big)$$
$$\le P\Big(\big|\mathbb{V} - \sqrt{n}(\widehat{\alpha}_n - \alpha_0)\big| \ge \epsilon, \omega \in \mathcal{E}\Big) \to 0, \text{ as } n \to \infty.$$

We conclude that

$$II_n = P\Big(\mathbb{V} \le t, \omega \in \mathcal{E}\Big) + o(1)$$

and

$$\limsup_{n\to\infty} \Big| P\Big(\sqrt{n}(\widehat{\alpha}_n - \alpha_0) \le t\Big) - P\Big(\mathbb{V} \le t, \omega \in \mathcal{E}\Big)\Big| \le \epsilon.$$

The latter implies that

$$\limsup_{n\to\infty} \Big| P\Big(\sqrt{n}(\widehat{\alpha}_n - \alpha_0) \le t\Big) - P(\mathbb{V} \le t) + P\Big(\mathbb{V} \le t, \omega \notin \mathcal{E}\Big)\Big| \le \epsilon$$

which, using the triangle inequality, yields

$$\limsup_{n\to\infty} \Big| P\Big(\sqrt{n}(\widehat{\alpha}_n - \alpha_0) \le t\Big) - P(\mathbb{V} \le t)\Big| \le \epsilon + P\Big(\mathbb{V} \le t, \omega \notin \mathcal{E}\Big)$$
$$\le 2\epsilon.$$

As ϵ can be made arbitrarily small, we conclude that $\lim_{n\to\infty} P\Big(\sqrt{n}(\widehat{\alpha}_n - \alpha_0) \le t\Big) = P(\mathbb{V} \le t)$. □

2 Proof of Theorem 3.1

Define

$$A_n(x) := \mathbb{F}_n(x) - (1 - \widehat{\alpha}_n)F_b(x),$$

so that

$$\widehat{a}_n = \sup\left\{x \geq \kappa_0 : A_n(x) \leq \widehat{\alpha}_n \frac{b_n}{\sqrt{n}}\right\}.$$

Note that

$$\begin{aligned} A_n(x) &= F_0(x) - (1-\alpha_0)F_b(x) + (\widehat{\alpha}_n - \alpha_0)F_b(x) + (\mathbb{F}_n(x) - F_0(x)) \\ &= \alpha_0 F_s(x) + (\widehat{\alpha}_n - \alpha_0)F_b(x) + (\mathbb{F}_n(x) - F_0(x)) \end{aligned}$$

Thus, for all $x \geq \kappa_0$, we have

$$\alpha_0 F_s(x) - |\widehat{\alpha}_n - \alpha_0| - \|\mathbb{F}_n - F_0\|_\infty \leq A_n(x) \leq \alpha_0 F_s(x) + |\widehat{\alpha}_n - \alpha_0| + \|\mathbb{F}_n - F_0\|_\infty$$

Hence, for all $x \geq \kappa_0$, we obtain with $d_n := \sqrt{n}|\widehat{\alpha}_n - \alpha_0| + \sqrt{n}\|\mathbb{F}_n - F_0\|_\infty$ that

$$\alpha_0 F_s(x) - \frac{d_n}{\sqrt{n}} \leq A_n(x) \leq \alpha_0 F_s(x) + \frac{d_n}{\sqrt{n}}. \tag{4}$$

Note that

$$\begin{aligned} \frac{1}{\alpha_0}\left(\widehat{\alpha}_n \frac{b_n}{\sqrt{n}} \pm \frac{d_n}{\sqrt{n}}\right) &= \frac{\widehat{\alpha}_n}{\alpha_0}\frac{b_n}{\sqrt{n}} \pm \frac{d_n}{\alpha_0\sqrt{n}} = \frac{b_n}{\sqrt{n}} + \frac{\widehat{\alpha}_n - \alpha_0}{\alpha_0}\frac{b_n}{\sqrt{n}} \pm \frac{d_n}{\alpha_0\sqrt{n}} \\ &= \frac{b_n}{\sqrt{n}}\left(1 + \frac{1}{\alpha_0}(\widehat{\alpha}_n - \alpha_0) \pm \frac{1}{\alpha_0}\frac{d_n}{b_n}\right) = \frac{b_n}{\sqrt{n}}\Big(1 + o_{\mathbb{P}}(1)\Big), \end{aligned}$$

where in the last step we applied Theorem 2.1, classical asymptotic properties of the empirical cdf (using Assumption A1) and also the condition that $b_n \nearrow \infty$. For n sufficiently large, $b_n/\sqrt{n} \searrow 0$ by assumption. Hence, for n large enough, there exists a unique $\breve{a}_n$ such that

$$F_s(\breve{a}_n) = \frac{1}{\alpha_0}\left(\widehat{\alpha}_n \frac{b_n}{\sqrt{n}} - \frac{d_n}{\sqrt{n}}\right) = \frac{b_n}{\sqrt{n}}\Big(1 + o_{\mathbb{P}}(1)\Big).$$

By a similar argument, there also exists a unique $\tilde{a}_n$ such that

$$F_s(\tilde{a}_n) = \frac{1}{\alpha_0}\left(\widehat{\alpha}_n \frac{b_n}{\sqrt{n}} + \frac{d_n}{\sqrt{n}}\right) = \frac{b_n}{\sqrt{n}}\Big(1 + o_{\mathbb{P}}(1)\Big).$$

But now, by Equation (4), we know that $\widehat{a}_n \in [\breve{a}_n, \tilde{a}_n]$, which implies that also

$$F_s(\widehat{a}_n) = \frac{b_n}{\sqrt{n}}\Big(1 + o_{\mathbb{P}}(1)\Big).$$

For n large enough, the right-hand side is strictly positive. This implies $\widehat{a}_n > a_0$. Using again the fact that $b_n/\sqrt{n} \searrow 0$, it follows that for n large enough, $F_s(\widehat{a}_n) < F_s(a_0+\eta)$. Since F_s is non-decreasing, we thus know that $\widehat{a}_n \in (a_0, a_0 + \eta)$ with increasing probability as n grows.

Let us now consider Case I. We suppose that F_s is differentiable on the whole interval $(a_0, a_0 + \eta)$. The right difference quotient at a_0, denoted by $f_s(a_0)$, is assumed to be non-zero and finite. By Taylor's theorem, we obtain

$$\begin{aligned}\frac{b_n}{\sqrt{n}}(1 + o_{\mathbb{P}}(1)) &= F_s(\widehat{a}_n) = F_s(a_0) + (\widehat{a}_n - a_0)f_s(a_0) + o(|\widehat{a}_n - \alpha_0|) \\ &= (\widehat{a}_n - a_0)(f_s(a_0) + o(1)).\end{aligned}$$

Hence,

$$\frac{\sqrt{n}}{b_n}\Big(\widehat{a}_n - \alpha_0\Big) = \frac{1 + o_{\mathbb{P}}(1)}{f_s(a_0) + o(1)} = \frac{1}{f_s(a_0)} + o_{\mathbb{P}}(1),$$

which is exactly what we wanted to show.
For Case II, fix an integer $k \geq 2$. We assume that F_s is $k-1$ times continuously differentiable on the interval $[a_0, a_0+\eta)$, with all the $k-1$ derivatives equal to zero. We also assume that the $(k-1)$-th derivative is continuously differentiable on the interval $(a_0, a_0+\eta)$ and that its right difference quotient exists at a_0, with the value $F_s^{(k)}(a_0)_+$ being non-zero (and necessarily strictly positive). Again by Taylor's theorem, we have that

$$\begin{aligned}\frac{b_n}{\sqrt{n}}(1 + o_{\mathbb{P}}(1)) &= F_s(\widehat{a}_n) = \frac{(\widehat{a}_n - a_0)^k}{k!} F_s^{(k)}(a_0)_+ + o(|\widehat{a}_n - a_0|^k) \\ &= (\widehat{a}_n - a_0)^k \left(\frac{F_s^{(k)}(a_0)_+}{k!} + o(1) \right),\end{aligned}$$

which implies

$$\left(\frac{\sqrt{n}}{b_n}\right)^{1/k}\Big(\widehat{a}_n - a_0\Big) = \left(\frac{1 + o_{\mathbb{P}}(1)}{\frac{F_s^{(k)}(a_0)_+}{k!} + o(1)} \right)^{1/k} = \left(\frac{k!}{F_s^{(k)}(a_0)_+} \right)^{1/k} + o_{\mathbb{P}}(1).$$

This yields the claimed convergence.
For the final Case III, we assume that F_s is differentiable on the whole interval $(a_0, a_0+\eta)$ but that the right difference quotient of F_s at a_0 is infinite. Formally, this means that

$$\lim_{t \downarrow a_0} \frac{F_s(t)}{t - a_0} = \lim_{t \downarrow a_0} \frac{F_s(t) - F_s(a_0)}{t - a_0} = \infty.$$

Let now $(u_n)_{n\geq 1}$ be an arbitrary non-negative null sequence. Then, there exists a sequence $(t_n)_{n\geq 1}$ such that for n large enough, we have $u_n = F_s(t_n)$, with $t_n \in (a_0, a_0+\eta)$, and on this interval, F_s^{-1} is well-defined. Thus,

$$\lim_{n\to\infty} \frac{F_s^{-1}(u_n) - a_0}{u_n} = \lim_{n\to\infty} \frac{F_s^{-1}(F_s(t_n)) - a_0}{F_s(t_n)} = \lim_{n\to\infty} \frac{t_n - a_0}{F_s(t_n)} = 0$$

by our assumption. But since $F_s^{-1}(0) = a_0$, the left-most expression is just the right difference quotient of F_s^{-1} at the point 0, and so this inverse function is right differentiable at 0 with derivative equal to 0. Applying now Taylor's theorem to F_s^{-1}, we get

$$\begin{aligned}\widehat{a}_n &= F_s^{-1}\left(\frac{b_n}{\sqrt{n}}(1+o_{\mathbb{P}}(1))\right) = F_s^{-1}(0) + \frac{b_n}{\sqrt{n}}(1+o_{\mathbb{P}}(1))\cdot 0 + o\left(\frac{b_n}{\sqrt{n}}(1+o_{\mathbb{P}}(1))\right)\\ &= a_0 + o_{\mathbb{P}}\left(\frac{b_n}{\sqrt{n}}\right),\end{aligned}$$

which implies that

$$\widehat{a}_n - a_0 = o_{\mathbb{P}}\left(\frac{b_n}{\sqrt{n}}\right),$$

and the proof is complete. □

Appendix A

Lemma A.1. *Let $\mathbb{B}$ denote again a standard Brownian Bridge. Then, the distribution of*

$$\mathbb{V} = -\inf_{[\kappa_0, a_0]} \frac{\mathbb{B}\circ F_0(x)}{F_b(x)}$$

is continuous.

Proof of Lemma A.1. Our goal is to show that $F(t) := \mathbb{P}(\mathbb{V}\leq t), t\in\mathbb{R}$ is continuous. We have

$$\begin{aligned}F(t) &= \mathbb{P}\left(-\inf_{[\kappa_0,a_0]} \frac{\mathbb{B}\circ F_0(x)}{F_b(x)} \leq t\right) = \mathbb{P}\left(\sup_{[\kappa_0,a_0]} -\frac{\mathbb{B}\circ F_0(x)}{F_b(x)} \leq t\right)\\ &= \mathbb{P}\left(\sup_{[\kappa_0,a_0]} \frac{(-\mathbb{B})\circ F_0(x)}{F_b(x)} \leq t\right) = \mathbb{P}\left(\sup_{[\kappa_0,a_0]} \frac{\mathbb{B}\circ F_0(x)}{F_b(x)} \leq t\right),\end{aligned}$$

using that $-\mathbb{B} = \mathbb{B}$ in distribution.
Define $\tilde{\mathcal{I}} := [\kappa_0, a_0]$. For all $x\in\tilde{\mathcal{I}}$, set $V(x) := (\mathbb{B}\circ F_0(x))/F_b(x)$. Note that for any fixed $x\in\tilde{\mathcal{I}}$, $V(x)$ is a Gaussian random variable. Also observe that by the above,

$F(t) = \mathbb{P}(V(x) \leq t, \forall x \in \tilde{\mathcal{I}})$. Define $\mathcal{I}$ to be the intersection of $\tilde{\mathcal{I}}$ with the rational numbers. We now show that in fact, we may restrict ourselves to $\mathcal{I}$.
Indeed, assume that for all $x \in \mathcal{I}$, we have $V(x) \leq t$. Choose now $y \in \tilde{\mathcal{I}} \setminus \mathbb{Q}$ arbitrarily. Since $\mathbb{Q}$ lies dense in $\mathbb{R}$, we know that there exists a sequence $(y_n)_{n \geq 1} \subseteq \mathcal{I}$ which converges to y. For the whole sequence, we have $V(y_n) \leq t$ by assumption. But now, by continuity of F_0 and F_b and the almost sure continuity of $\mathbb{B}$, we know that almost surely, $V(y_n) \to V(y)$. Hence, also $V(y) \leq t$. Since y was chosen arbitrarily, we thus know that $V(x) \leq t$ for all $x \in \tilde{\mathcal{I}}$. Hence, from now on, we are able to work with the countable set $\mathcal{I}$.
We have

$$\begin{aligned}
\{V(x) \leq t, \forall x \in \mathcal{I}\} \setminus \{V(x) < t, \forall x \in \mathcal{I}\} &= \{V(x) \leq t, \forall x \in \mathcal{I}\} \cap \{\exists x \in \mathcal{I} : V(x) \geq t\} \\
&= \{V(x) \leq t, \forall x \in \mathcal{I}, \exists x \in \mathcal{I} : V(x) = t\} \\
&\subseteq \{\exists x \in \mathcal{I} : V(x) = t\} \\
&= \bigcup_{x \in \mathcal{I}} \{V(x) = t\}
\end{aligned}$$

Since for all $x \in \mathcal{I}$, $V(x)$ is a Gaussian random variable, each of these events has probability zero. As $\mathcal{I}$ is countable, we thus have a countable union of negligible sets, which has still probability zero. In particular,

$$\mathbb{P}(V(x) \leq t, \forall x \in \mathcal{I}) - \mathbb{P}(V(x) < t, \forall x \in \mathcal{I}) = 0.$$

We deduce that F is continuous. □

Appendix B

References

Komlos, J., Major, P., Tusnády, G. (1975). An approximation of partial sums of independent rv'-s, and the sample df. i. *Probability Theory and Related Fields*, *32*, 111-131, https://doi.org/10.1007/BF00533093

Shorack, G.R., & Wellner, J.A. (2009). *Empirical processes with applications to statistics.* Philadelphia: Society for Industrial and Applied Mathematics.

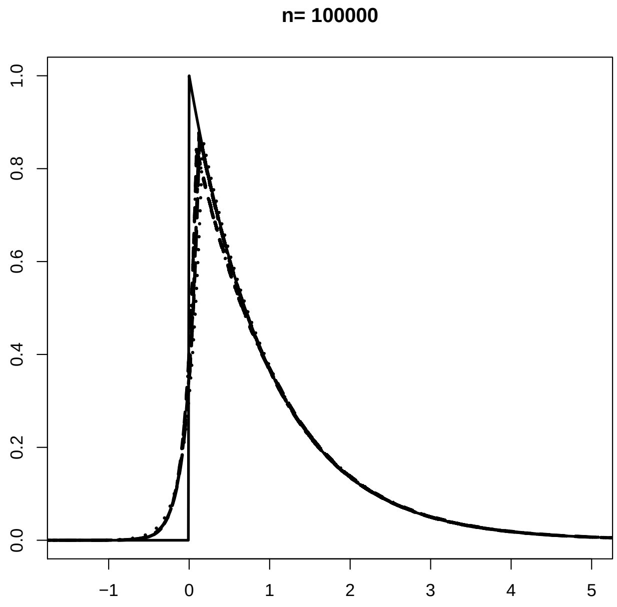

Fig. B1 The figure shows 5 (pseudo) log-concave MLE based on independent samples of size $n = 100000$ from Exp(1). The mixing proportion is $\alpha_0 = 0.95$.
